# Identification and validation of a glycolysis‐related taxonomy for improving outcomes in glioma

**DOI:** 10.1111/cns.14601

**Published:** 2024-02-08

**Authors:** Tianshu Ying, Yaming Lai, Shiyang Lu, Shaolong E

**Affiliations:** ^1^ Department of Oncology Shengjing Hospital of China Medical University Shenyang China; ^2^ Department of Urology Guangyuan Central Hospital Guangyuan China; ^3^ Department of Urology Shengjing Hospital of China Medical University Shenyang China

**Keywords:** ADM, glioma, glycolysis, ingenol mebutate, taxonomy

## Abstract

**Background:**

Reprogramming of glucose metabolism is a prominent abnormal energy metabolism in glioma. However, the efficacy of treatments targeting glycolysis varies among patients. The present study aimed to classify distinct glycolysis subtypes (GS) of glioma, which may help to improve the therapy response.

**Methods:**

The expression profiles of glioma were downloaded from public datasets to perform an enhanced clustering analysis to determine the GS. A total of 101 combinations based on 10 machine learning algorithms were performed to screen out the most valuable glycolysis‐related glioma signature (GGS). Through RSF and plsRcox algorithms, adrenomedullin (ADM) was eventually obtained as the most significant glycolysis‐related gene for prognostic prediction in glioma. Furthermore, drug sensitivity analysis, molecular docking, and in vitro experiments were utilized to verify the efficacy of ADM and ingenol mebutate (IM).

**Results:**

Glioma patients were classified into five distinct GS (GS1‐GS5), characterized by varying glycolytic metabolism levels, molecular expression, immune cell infiltration, immunogenic modulators, and clinical features. Anti‐CTLA4 and anti‐PD‐L1 antibodies significantly improved the prognosis for GS2 and GS5, respectively. ADM has been identified as a potential biomarker for targeted glycolytic therapy in glioma patients. In vitro experiments demonstrated that IM inhibited glioma cell progression by inhibiting ADM.

**Conclusion:**

This study elucidates that evaluating GS is essential for comprehending the heterogeneity of glioma, which is pivotal for predicting immune cell infiltration (ICI) characterization, prognosis, and personalized immunotherapy regimens. We also explored the glycolysis‐related genes ADM and IM to develop a theoretical framework for anti‐tumor strategies targeting glycolysis.

## Graphical abstract

We constructed a glycolysis‐related taxonomy for improving outcomes in glioma. We also explored the glycolysis‐related gene ADM and ingenol mebutate to provide a theoretical framework for anti‐tumor strategy targeting glycolysis.

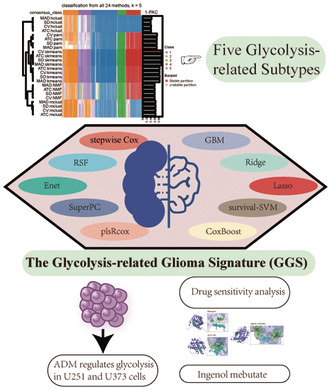

## 1 BACKGROUND

Glioma is the most common and lethal primary brain tumor in adults, affecting the central nervous system (CNS). It has a dismal prognosis and can progress to become untreatable and resistant to chemoradiation therapy.^1^, ^2^ Glioma is characterized by a high rate of recurrence and metastasis, with a median survival time of less than 1.5 years for patients.^3^, ^4^

A diagnostic framework based on the molecular and genetic characteristics of CNS tumors was adopted in the WHO CNS tumor classification update.^5^ In addition, a layered reporting system consisting of integrated diagnosis, histologic diagnosis, WHO classification, and molecular information was incorporated. For glioma molecular classification and prognostication, various molecular abnormalities have received considerable attention, including IDH1/2 mutations, ATRX mutations, 1p/19q co‐deletion, TERT promoter mutations, and MGMT promoter methylation.^6^ Despite their effectiveness in glioma classification, the 5‐year relative survival rates for glioma in the USA are less than 5%.^7^ Therefore, relying solely on histopathology or a single gene modification may not be sufficient for reliable glioma classification. It is crucial to investigate the molecular features of glioma and identify biomarkers that can accurately predict treatment response.

Glioma is characterized by altered cellular metabolism,^8^ with increased glycolysis being the most well‐known metabolic alteration associated with tumor cells.^9^ This phenomenon, known as the Warburg effect, occurs even in the presence of sufficient oxygen, leading to a preference for glycolysis over oxidative phosphorylation by the majority of tumor cells.^10^ Previous research by Xin Chen et al. demonstrated that HLA‐F stimulated HK2‐dependent glycolysis, thereby upregulating glioma cell proliferation.^11^ As such, investigations into glycolysis may pave the way for a new classification system for glioma and the identification of novel therapeutic targets.

We identified and diversely validated five glycolysis subtypes (GS; GS1‐GS5) with unique clinical and biochemical peculiarities using machine learning‐based integration. Our findings increased the understanding of glioma heterogeneity from the glycolysis perspective and offered a high‐resolution taxonomy.

## 2 MATERIALS AND METHODS

### 2.1 Clinical specimens

The glioma and normal brain specimens utilized in the study were obtained from Shengjing Hospital Affiliated to China Medical University. The medical Ethics Committee of Shengjing Hospital Affiliated to China Medical University gave ethical approval to the study.

### 2.2 Data source

Data were collected from various public databases, including The Cancer Genome Atlas (TCGA‐GBM), Chinese Glioma Genome Atlas (CGGA‐693 and CGGA‐325), Rembrandt, Gorovets, Gravendel, Grzmil, Ducray, Murat, Kamoun, POLA Network, and Vital datasets. All datasets were downloaded from GlioVis,^12^ and the corresponding websites were listed in Table S1. To meet the statistical requirements of the subsequent study, we integrated the clinical data, which included gender, age, grade, survival status, and survival duration (in days). Default values were removed during this process.

A total of 289 glycolysis‐related genes were obtained from HALLMARKER, KEGG_GLYCOLYSIS_GLUCONEOGENESIS, REACTOME_GLYCOLYSIS, WP_GLYCOLYSIS_AND_GLUCONEOGENESIS in the Molecular Signatures Database.

### 2.3 Consensus clustering

Using a predefined set of glycolysis‐related genes, we applied 24 top‐genes/clustering approaches to identify stable gene expression subtypes. We reduced dimensionality using the Rtsne (v0.15) algorithm and DESeq2 VST data matrix, and visualized the projected GS using t‐distributed Stochastic Neighbor Embedding (t‐SNE) and the properties of five unsupervised clusters.

### 2.4 Machine learning‐based integrative approaches

Prior to employing machine learning techniques, univariate Cox regression analysis was utilized to identify prognosis‐related genes. A total of 101 distinct models were examined, including Elastic network [Enet], Stepwise Cox, partial least squares regression for Cox [plsRcox], Random survival forest [RSF], Supervised principal components [SuperPC], CoxBoost, Lasso, survival support vector machine [survival‐SVM], Ridge, and Generalized boosted regression modeling [GBM]. The optimal model combination was selected based on the highest C‐index across eight distinct datasets.^13^

### 2.5 Drug sensitivity prediction and molecular docking

Drug sensitivity data was collected using the CTRP v.2.0 and PRISM Repurposing datasets. Drug sensitivity was estimated using the AUC values from both datasets. A lower AUC value indicates increased sensitivity to a specific drug. Missing AUC values were imputed using KNN imputation, employing the closest neighbor algorithm. Compounds with >20% missing data were disregarded before imputation. Molecular docking models of adrenomedullin (ADM)‐compound binding were generated using AutoDock and PyMol.

### 2.6 Cell culture and treatment

Human glioma cell lines (U87, U251, U373, and A172) were purchased from the Shanghai Institutes for Biological Sciences Cell Resource Center and cultured in DMEM (HyClone, Logan, UT) supplemented with 10% FBS (TBD, Tianjin, China) following standardized protocols. Normal human astrocytes (NHA) were bought from ScienCell Research Laboratories, which were cultivated in astrocyte medium (ScienCell, Carlsbad, CA). All cells were grown at 37°C in an incubator with 5% CO_2_ humidity and cells (5× 10^4^ per well) were exposed to 10 μM ingenol mebutate (HY‐B0719) for 48 h.^14^, ^15^ IM‐treated cells received the same amounts of the solvent DMSO as control cells.

### 2.7 Cell transfection

Cells were seeded into 24‐well plates and Lipofectamine3000 reagent (Life Technologies, USA) was used to transfect cells with plasmids when cell confluence reached 50%–70%. ADM full‐length plasmid ADM (+), short‐hairpin ADM (−) and their respective negative control plasmid (ADM (−) NC and ADM (+) NC) were synthesized by GeneChem (China). G418 was used to screen the stably transfected cell. The shRNA sequences were listed in Table S2.

### 2.8 qRT‐PCR

Trizol (Invitrogen, USA) was used to extract the RNA from tissues and cells. SYBR Green Kit (Vazyme, China) was used for qRT‐PCR according to the protocol. β‐actin was used as internal reference. Table S3 presented the primers that were used.

### 2.9 Western blot analysis

After using the BCA method to determine the protein concentration, protein samples were added to each well and separated by SDS‐PAGE. Proteins were transferred to polyvinylidene fluoride membranes after SDS‐PAGE electrophoresis. Following that, membranes were blocked in 5% nonfat milk for 1 h at room temperature. The membranes were incubated in the appropriate primary antibody overnight at 4°C. The following were the primary antibodies: ADM (Proteintech, USA) and β‐actin (Proteintech, USA).

### 2.10 Transwell assay

The ability of U373 and U251 cells to migrate and invade was evaluated using transwell chambers (#3422 Costar, Corning, NY, USA). Glioma cells (2 × 10^4^) with serum‐free culture medium were seeded into upper chambers. 10% FBS medium was put into the lower chamber. For the cell invasion assay, upper chambers were precoated with Matrigel (BD, Franklin Lakes, NJ, USA) before cells being seeded into the top chamber. Cells that had migrated or invaded to the lower side of the membrane were fixed and then stained with 10% Giemsa after 24 h of incubation. Cells could be counted and photographed under a microscope.

### 2.11 Cell viability assay

The cell viability was assessed using the Cell Counting Kit‐8 (Dojindo, Kumamoto, Japan) following the manufacturer's guidelines. Cells were seeded into 96‐well plates and incubated for 48 h at 37°C. After adding 10 μL of CCK‐8 reagent to each well, the plate was incubated for 2 h at 37°C. The absorbance was measured at 450 nm.

### 2.12 Glucose and lactate measurement and Seahorse XF glycolysis stress assay

Using the corresponding kits (Nanjing Jiancheng, Jiangsu, China), the concentrations of lactate and glucose were determined according to the manufacturer's instructions. Using XF24 Extracellular Flux Analyzer and Seahorse XF Glycolysis Stress Test Kit (Seahorse Bioscience, MA, USA), the extracellular acidification rate (ECAR) was measured.

### 2.13 Other bioinformatics analysis and statistical analysis

Subclass mapping (SubMap) methodology was used to assess the consistency of glioma clustering of NTP and PAM methodologies.^16^ Silhouette scores were determined using the specified clusters.^17^ Enrichment pathways were identified using the R package clusterProfiler and Gene Set Variation Analysis (GSVA).^18^ Enrichment scores were assessed using the GSVA R package's single‐sample Gene Set Enrichment Analysis (ssGSEA) function. To evaluate immune cell infiltration abundances, five additional approaches were applied: Cibersort, xCell, MCPcounter, EPIC, and QuanTIseq.

The data are presented as the mean ± SD. The Shapiro–Wilk test was utilized to assess the normality of the data. One‐way ANOVA and Student's *t*‐tests were performed for data with a normal distribution. For data that did not conform to a normal distribution, the nonparametric test was applied. All analyses were conducted using either GraphPad Prism 8 or R software version 4.0.2, with *p* < 0.05 considered statistically significant.

## 3 RESULTS

### 3.1 Development and validation of glycolysis related consensus clusters

**FIGURE 1:**
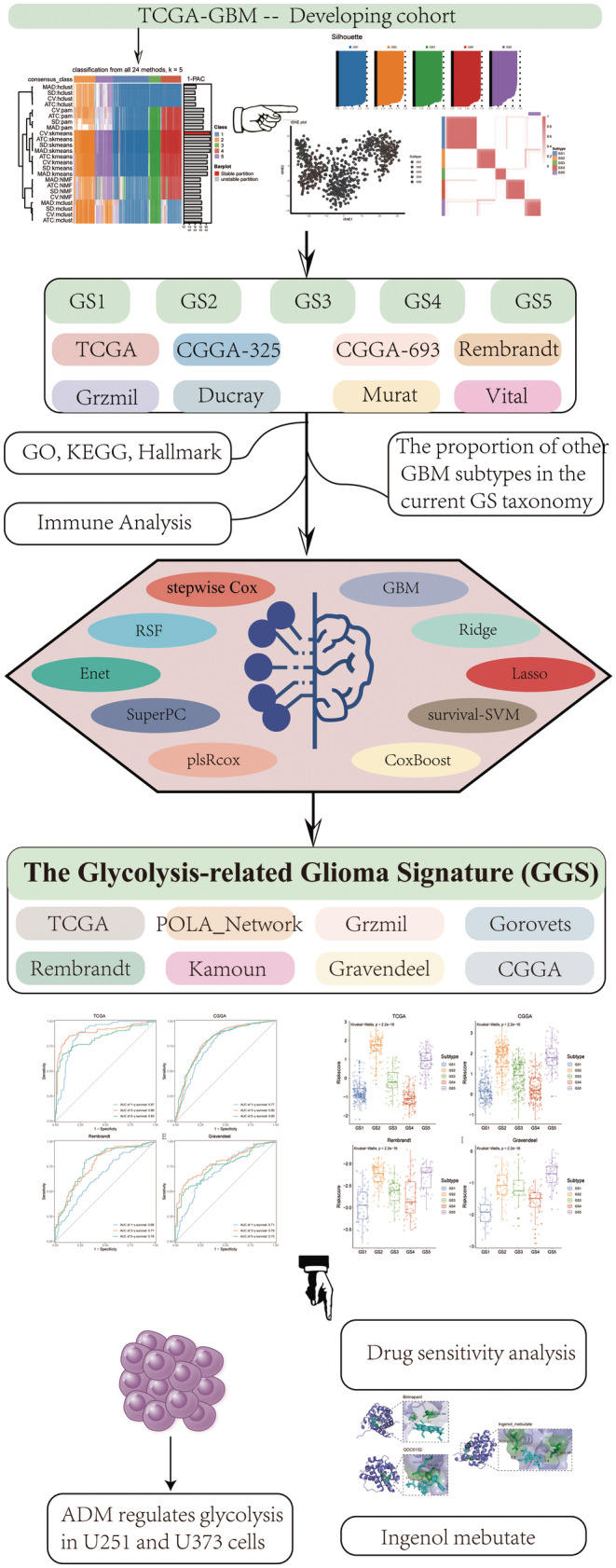
The workflow of our research.

**FIGURE 2:**
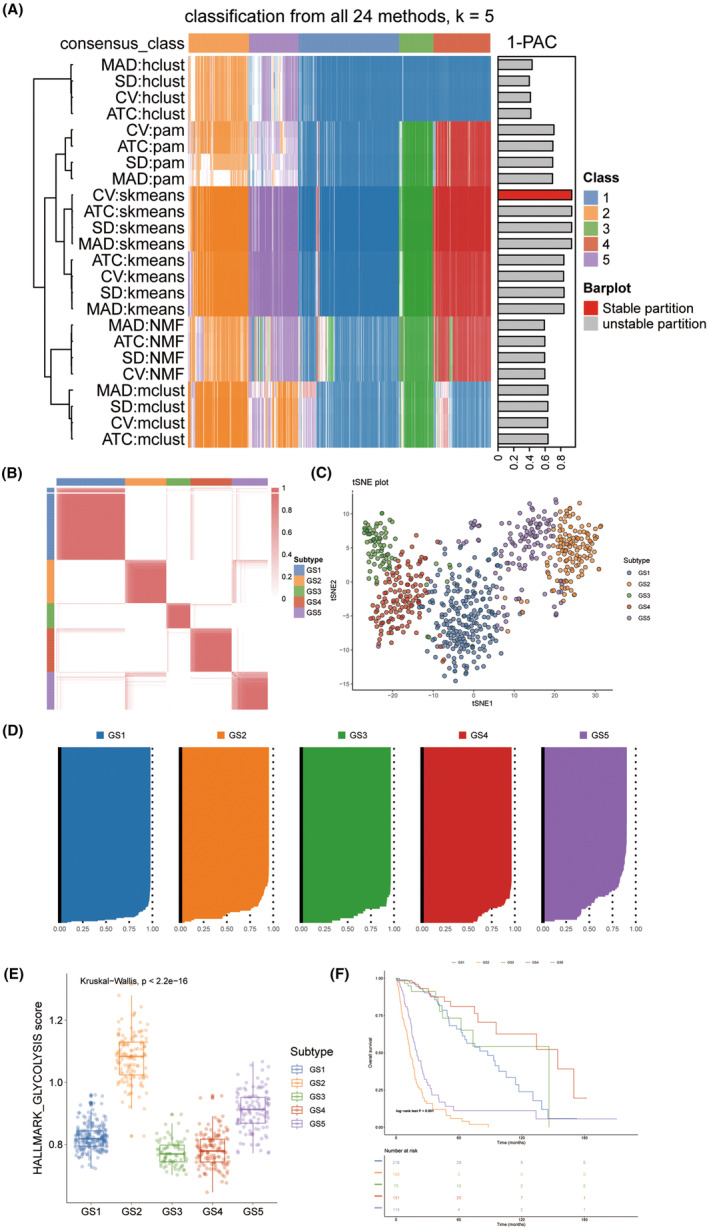
Characteristic glioma subtypes defined by glycolysis‐related gene expression profiling. (A) Consensus clustering (membership heatmap) of gene expression profiling from glioma patients with the use of 24 different computational parameters. The PAC negatively indicated the stability of clustering results (Left). Heatmap displayed representative consensus clustering method (skmeans) based on top variance genes, which showed the consistency of two samples in the same subtype (Right). (B) The consensus score matrix of all samples when *K* achieved 5. A higher consensus score between two samples indicated they were more likely to be grouped into the same cluster in different iterations. (C) The tSNE analysis cast all samples in two‐dimensional spatial coordinates, showing good discrimination. (D) Silhouette width of samples in each subtype. (E) The distribution of glycolysis score through ssGSEA in five GS. (F) The Kaplan–Meier curves of overall survival with log‐rank test for five GS. Log‐rank test.

Figure 1 provided a detailed overview of the workflow. Using an enhanced consensus clustering approach on RNA‐Seq data, including 24 top clustering methods, we selected 289 glycolysis‐related genes and identified five stable subtypes of glioma (GS1‐GS5) (Figure 2A). In addition, consensus clustering analysis,^19^ t‐SNE,^20^ and silhouette statistics all demonstrated high accuracy in five stable subtypes of glioma (Figure 2B–D). GS varied significantly in survival according to the Kaplan–Meier analysis. The overall survival (OS) predictions for GS2 and GS5 were the poorest, while GS3 and GS4 had the most promising outcomes, with GS1 exhibiting moderate OS (*p* < 0.001; Figure 2E). We then examined the disparities in glycolysis metabolism among the five GS using ssGSEA (Figure 2F). Significantly, glioma patients consistently experienced worse outcomes when their glycolysis metabolism levels were higher.

### 3.2 Evaluation of the glycolysis‐related taxonomy

**FIGURE 3:**
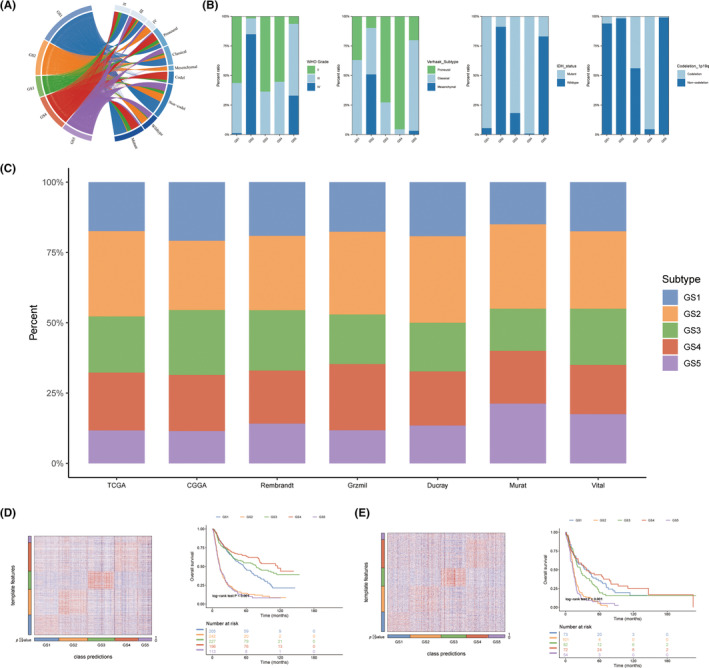
Five subtypes were reproductive and stable in multiple datasets. (A) Chord diagram showed connections among five GS, WHO classification, Verhaak Subtype, IDH status, and 1p/19q co‐deletion. (B) Barplots showed the proportion of other glioma subtypes (WHO classification, Verhaak Subtype, IDH status, and 1q19p co‐deletion) in the current five GS taxonomy. (C) Barplots showed comparable fractions of patients being assigned to each subtype in TCGA, CGGA, Rembrandt, Grzmil, Ducray, Murat, and Vital datasets. (D‐E) Two datasets, CGGA and Rembrandt, were assigned in five subtypes according to the classifier. The bottom and left bars indicated the subtypes. In the heatmap, rows indicated genes from the classifier and columns represent patients. The heatmap was color‐coded on the basis of median‐centered log_2_ gene expression levels (red, high expression; blue, low expression). The Kaplan–Meier curves of the overall survival with log‐rank test corresponding subtypes in CGGA and Rembrandt cohorts, located in the right panel.

The novel GS identified by the WHO classification, Verhaak Subtype, IDH status, and 1q/19p subtype were strongly correlated with glioma entities (Figure 3A,B). The GS2 and GS5 closely resembled WHO IV grade, IDH wildtype, mesenchymal, and 1p/19q co‐deletion tumors, which were more aggressive, prone to metastasis, and had the worst prognosis. In contrast, the GS4 predominantly harbored favorable genetic lesions such as IDH mutation, 1p/19q co‐deletion, and more proneural tissue, resulting in the most favorable prognosis. Across all eight datasets, five GS exhibited analogous transcriptional and clinical features, as well as similar proportions (Figure 3C). Furthermore, the molecular characteristics and overall survival of CGGA and Rembrandt datasets were comparable (Figure 3D,E). These findings underscored the accuracy and stability of glioma GS.

### 3.3 Mfuzz cluster and bioinformatic analyses for GS

**FIGURE 4:**
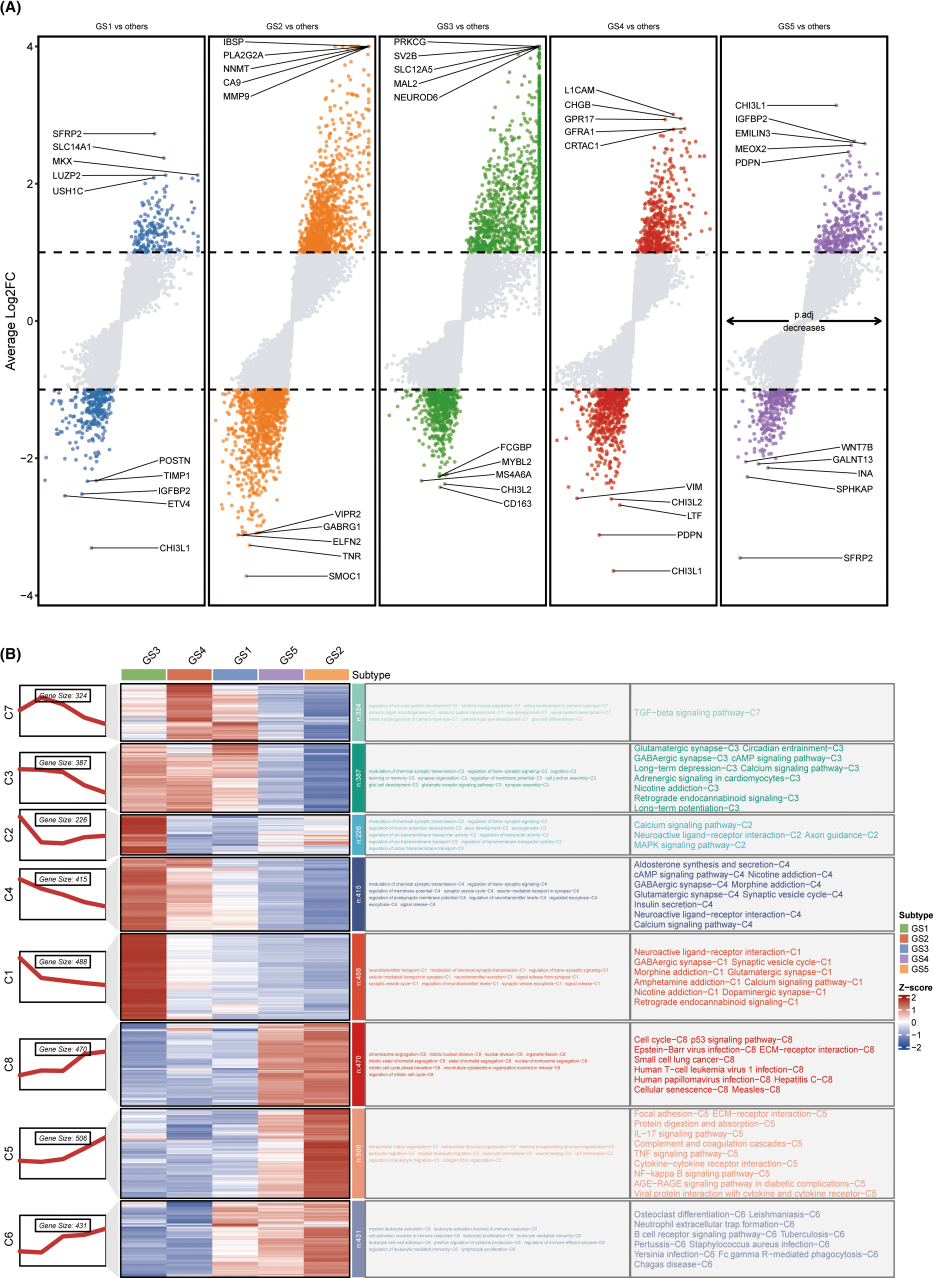
Molecular features of glycolysis‐related gene expression subtypes. (A) Scatter plots indicates the top upregulated and downregulated genes in five GS. Different GS are labeled by different colors. The *y*‐axis indicates log_2_FC of gene expressions versus rest samples. (B) Eight gene clusters were obtained via the soft clustering method (Mfuzz) in GS. The expression distribution of eight gene clusters among five GS. GO and KEGG‐based analysis delineated the biological attributes inherent to GS.

In comparison to other glioma subtypes, the GS2 exhibited higher expression levels of IBSP, PLA2G2A, NNMT, CA9, and MMP9, while the GS5 showed higher expression levels of CHI3L1, IGFBP2, EMILIN3, MEOX2, and PDPN. Genes highly expressed in these two subtypes may have oncogenic effects. Conversely, the GS1/3/4 subtypes displayed higher expression levels of SFRP2, SLC14A1, MKX, LUZP2, and USH1C; PRKCG, SV2B, SLC12A5, MAL2, and NEUROD6; L1CAM, CHGB, GPR17, GFRA1, and CRTAC1, respectively (Figure 4A). To identify the genes essential for prognostic variations in GS, we employed the Mfuzz package^21^ to examine the expression trends of differentially expressed genes (DEGs). This analysis revealed eight clusters that were subsequently divided into two groups: the upregulated group (C5, C6, and C8) and the downregulated group (C1‐4 and C7). The upregulated group displayed a direct correlation between increased expression and more severe GS (Figure 4B). Gene ontology (GO) and Kyoto Encyclopedia of Genes and Genomes (KEGG) enrichment analyses indicated that the upregulated group was significantly enriched in oncogenic pathways such as cell cycle‐related pathways, p53 signaling pathway, focal adhesion, and extracellular matrix (ECM) − receptor interaction, as well as immune‐related pathways (Figure 4B).

**FIGURE 5:**
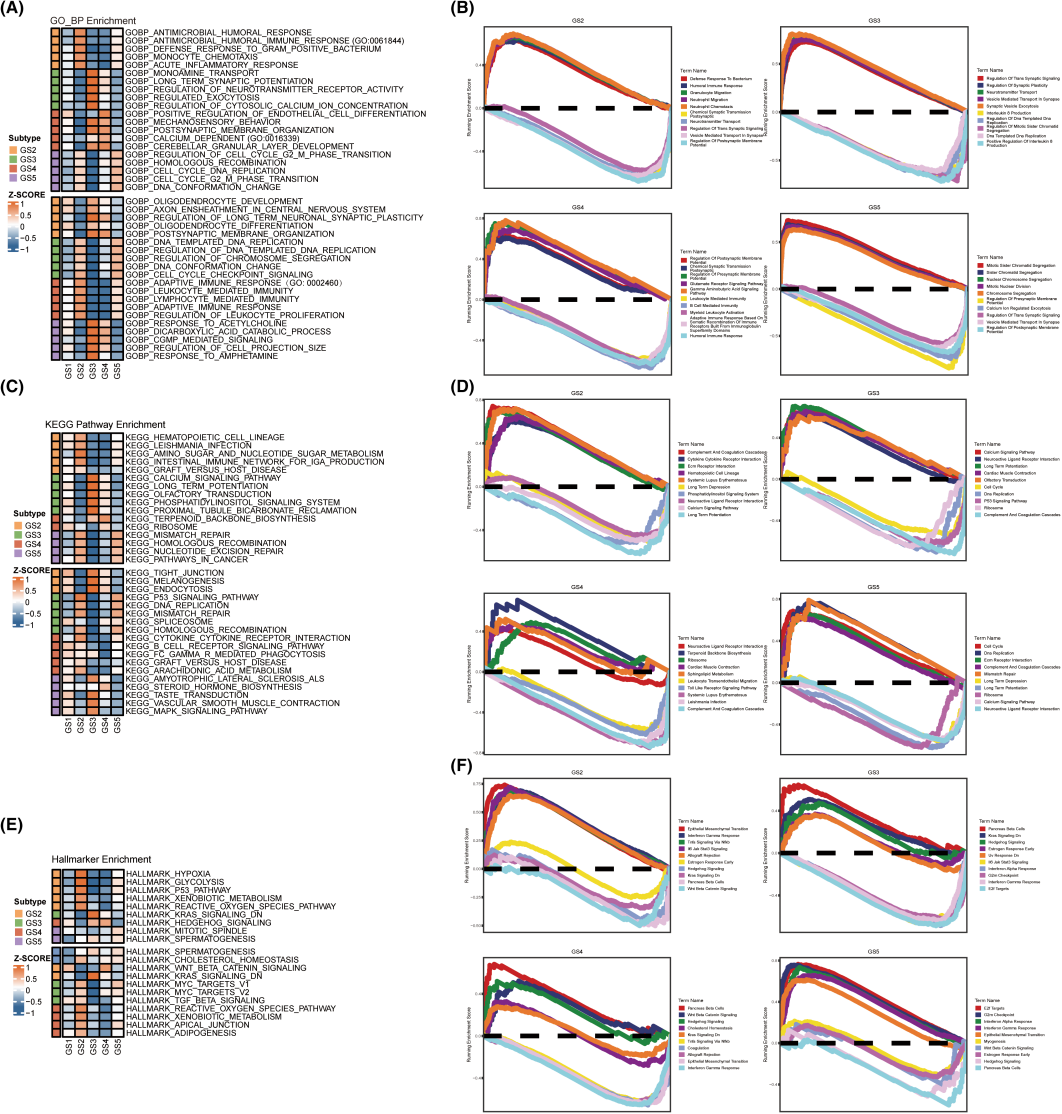
Biological peculiarities of five subtypes. (A, C, E) GSVA‐based analysis delineated the biological attributes inherent to GS in the Gene ontology (GO), Kyoto Encyclopedia of Genes and Genomes (KEGG), and Hallmark (GSEA)‐enriched pathways‐enriched pathways. (B) The Gene ontology (GO)‐enriched pathways in four glycolysis subtypes (GS2‐GS5). (D) The Kyoto Encyclopedia of Genes and Genomes (KEGG)‐enriched pathways in GS. (F) The Hallmark (GSEA)‐enriched pathways in GS.

The potential functional pathways of various GS were elucidated using gene set enrichment analysis (GSEA). As shown in Figure 5, GS1 was distinguished by regulation of nervous system development, glial cell differentiation and development, glutamate receptor signaling pathway, TGF‐β signaling pathway, circadian entrainment, leukocyte proliferation, cell activation involved in immune response, and B cell receptor signaling pathway. GS2 was significantly enriched for pathways related to the immune system and metabolism, including amino sugar and nucleotide sugar metabolism, hypoxia, glycolysis, P53 pathway, oxidative stress, monocyte chemotaxis, humoral response, and acute inflammatory response. In contrast, KRAS signaling and Wnt‐β catenin signaling were suppressed. GS5 was prominently enriched for DNA repair and cell cycle pathways, which contributed to its higher grades and worse prognosis. These pathways included homologous recombination, DNA conformation change, mismatch repair, nucleotide excision repair, cell cycle DNA replication, cell cycle G2M phase transition, mitotic spindle, and cancer‐related pathways. In contrast, MAPK signaling pathway and CGMP‐mediated signaling was inhibited.

GS3 exhibited a significant enrichment of the KRAS signaling pathway and a negative correlation with cell cycle and DNA repair processes, which could account for its lower grade and moderate prognosis. In contrast, GS4 displayed an overrepresentation of hedgehog signaling, calcium‐dependent cell adhesion through plasma membrane cell adhesion molecules, terpene skeleton biosynthesis, and a predominantly negative association with immune‐related pathways, including adaptive immune response, leukocyte‐mediated immunity, lymphocyte‐mediated immunity, B‐cell receptor signaling pathway, and leukocyte proliferation.

### 3.4 Immune landscape

**FIGURE 6:**
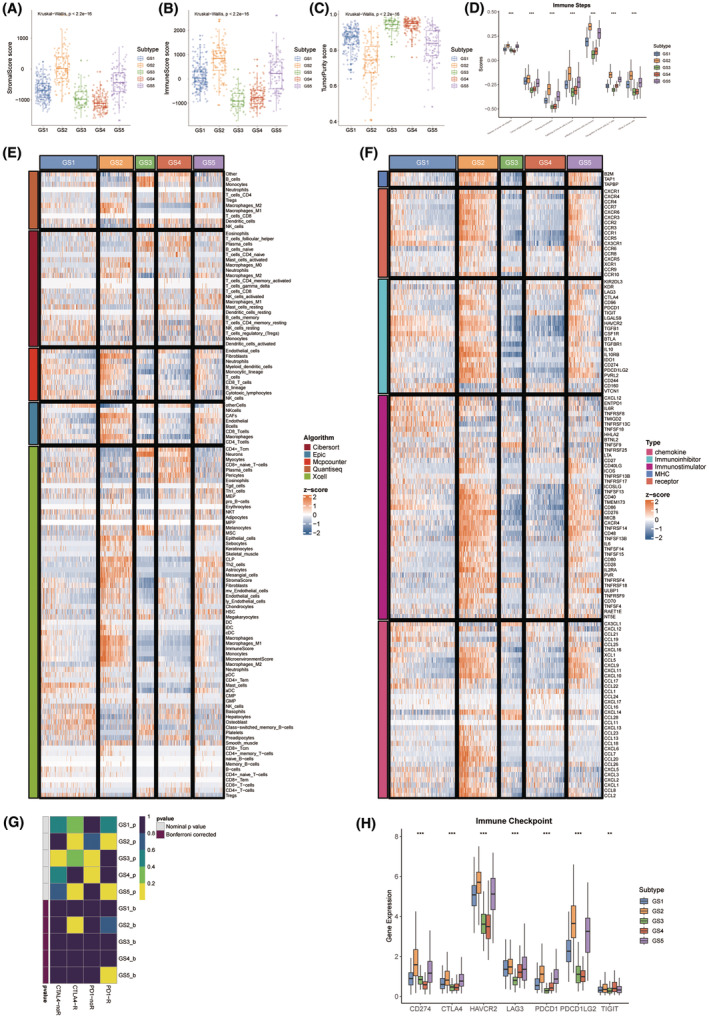
Immune landscape and immunotherapeutic potential of five subtypes. (A) The distribution of stromal score in different GS. (B) The distribution of immune score in different GS. (C) The distribution of tumor purity score in different GS. (D) Boxplot showed autocrine stimulation loops in GS. (E) The differences in infiltration profiles of multiple immune cells between five GS. The infiltration profiles were evaluated by several algorithms including CIBERSORT, EPIC, MCP‐counter, quanTIseq, and xCell, and then normalized and scaled into *Z*‐score. (F) The differences in expression profiles of immune regulators between five GS. The list of immune regulators was downloaded from TISIDB (http://cis.hku.hk/TISIDB/). (G) SubMap module of GenePattern predicted the responses to anti‐PD‐1 and anti‐CTLA‐4 between different GS. (H) The distribution of gene expression of ICIs in different GS. *p < 0.05; **p < 0.01.

The presence of substantial tumor tissue was observed in GS3 and GS4, while GS2 and GS5 exhibited a higher density of stromal cells and infiltrating immune cells (Figure 6A–C). Consistent results were obtained when employing the Cancer Immune Cycle (CIC) boxplot and various Tumor micro‐environment (TME) contexture decoding algorithms (Figure 6D,E). In this context, GS2 and GS5 were classified as “hot” immune tumors, while GS3 and GS4 were categorized as “cold” immune tumors.^22^ Furthermore, a consistent pattern was observed in the expression profiles of immune regulators (Figure 6F). The SubMap analysis revealed a comparable expression pattern between GS2 and anti‐CTLA‐4 responders, as well as between GS5 and anti‐PD‐1 responders (Figure 6G). Similar trends were also seen in immune checkpoint expression (Figure 6H). In summary, immunotherapy may yield clinical benefits for patients with GS2 and GS5 tumors, while those with GS1, GS3, and GS4 tumors may not be suitable candidates due to potential immune‐related adverse events or high costs.

### 3.5 Construction of glycolysis‐related glioma signature (GGS)

**FIGURE 7:**
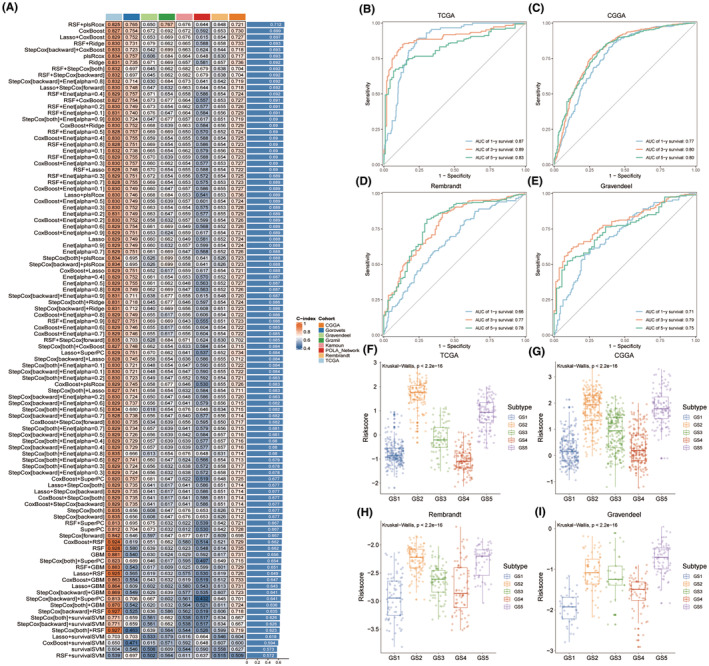
Construction and testing of the glycolysis‐related glioma signature (GGS). (A) The hub genes were developed using a total of 101 machine‐learning combinations via a 10‐fold cross‐validation framework. The c‐index of each combination was evaluated in all datasets. (B‐E) Time‐dependent ROC analysis revealed the AUC of hub genes for 1‐, 3‐, and 5‐year OS in TCGA‐GBM, CGGA, Rembrandt, and Gravendeel datasets. (F‐I) The distribution of riskscore between five GS in TCGA‐GBM, CGGA, Rembrandt, and Gravendeel datasets.

We employed the 101 advanced machine learning methods to identify the most robust glycolysis‐related signature with the highest C‐index in nine cohorts (CGGA‐325, CGGA‐693, Gorovets, Gravendeel, Grzmil, Kamoun, POLA Network, Rembrandt, and TCGA) (Figure 7A). A final glycolysis‐related signature with optimal performance was established using the combined RSF and plsRcox algorithm (*C*‐index = 0.712). After downscaling the model using this algorithm, we selected 41 genes.

Based on the median GGS riskscore, glioma patients were divided into high‐GGS and low‐GGS groups. The discriminatory power of the GGS riskscore in terms of survival was evaluated using time‐dependent ROC analysis (Figure 7B–E; Figure S1B,D,F,H). The results consistently showed that the GGS riskscore performed well across several datasets. Furthermore, multiple datasets (TCGA, CGGA, Rembrandt, and Gravendeel) revealed that glioma patients with higher GGS riskscores exhibited increased glycolysis metabolism and shorter survival times (Figures 2E and 7F–I; Figure S1A,C,E,G).

### 3.6 ADM overexpression promotes glycolysis and proliferation in glioma cells

**FIGURE 8:**
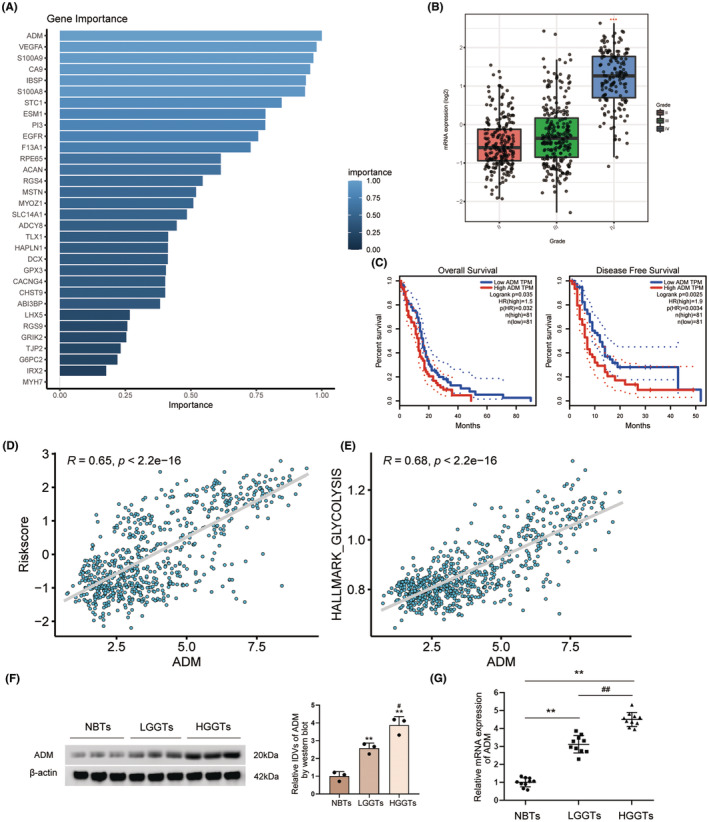
The expression of ADM in glioma. (A) Histogram showed that importance of selected hub genes. (B) The mRNA expression of ADM between the grade levels. (C) High expression of ADM was related to decreased OS and DFS. Survival analysis was employed by the Kaplan–Meier curves with the log‐rank test. (D) Pearson's correlation between riskscore and the expression of ADM. (E) Pearson's correlation between glycolysis levels and the expression of ADM. (F) ADM protein expression in NBTs as well as LGGTs and HGGTs groups were detected using western blot. The data for each group are presented as mean ± SD (*n* = 3 for each group). **p < 0.01 versus NBTs group; ^#^ *p* < 0.05 versus LGGTs group. (G) The expression level of ADM mRNA in the NBTs, LGGTs, and HGGTs groups were detected via qRT‐PCR. The data for each group are presented as mean ± SD (*n* = 10, each group); **p < 0.01 versus NBTs group; ^##^ *p* < 0.01 versus LGGTs group.

Through RSF + plsRcox algorithms, we obtained the weight for 41 genes to characterize the importance of the gene to the prognosis of glioma. ADM was eventually obtained as the most significant glycolysis‐related gene for prognostic prediction in glioma (Figure 8A).

**FIGURE 9:**
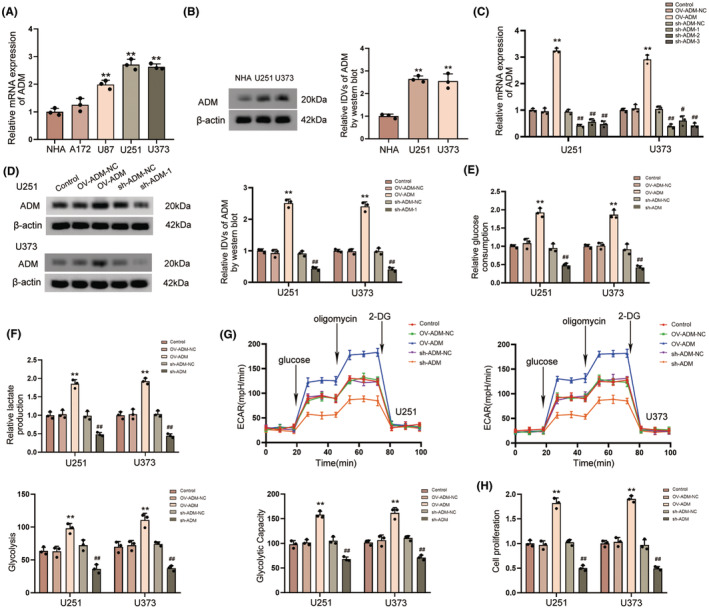
The effect of ADM on glycolysis and proliferation in glioma. (A) The expression of ADM mRNA in NHA, U87, U251, U373, and A172 cells. The data for each group are presented as mean ± SD (*n* = 3, each group); **p < 0.01 versus NHA group. (B) The expression of ADM protein in NHA and glioma cell lines (U251 and U373). The data for each group are presented as mean ± SD (*n* = 3, each group); **p < 0.01 versus NHA group. (C) Transfection efficiency of ADM in U251 and U373 cells detected by qRT–PCR. The data for each group are presented as mean ± SD (*n* = 3 for each group); **p < 0.01 versus OV‐ADM‐NC group; ^#^ *p* < 0.05, ^##^ *p* < 0.01 versus sh‐ADM‐NC group. (D) Transfection efficiency of ADM in U251 and U373 cells detected by western blot. Data are shown as the mean ± SD (*n* = 3 for each group); **p < 0.01 versus OV‐ADM‐NC group; ^##^ *p* < 0.01 versus sh‐ADM‐NC group. (E, F) The effects of ADM on glucose consumption (E) and lactate production (F) in U251 and U373 cells; Data are shown as the mean ± SD (*n* = 3, each group). **p < 0.01 versus OV‐ADM‐NC group; ^##^ *p* < 0.01 versus sh‐ADM‐NC group. (G) The effects of ADM overexpression and knockdown on ECAR in U251 and U373 cells were detected, and the glycolysis and glycolytic capability were analyzed; Data are presented as the mean ± SD (*n* = 3, each group). **p < 0.01 versus OV‐ADM‐NC group; ^##^ *p* < 0.01 versus sh‐ADM‐NC group. (H) Cell viability assays were used to detect the effects of ADM on proliferation in U251 and U373 cells. Data are shown as the mean ± SD (*n* = 3, each group); **p < 0.01 versus OV‐ADM‐NC group; ^##^ *p* < 0.01 versus sh‐ADM‐NC.

With increasing glioma grade, the mRNA expression levels of ADM were found to be upregulated (Figure 8B). The GEPIA database^6^ revealed a negative correlation between ADM expression and OS as well as DFS in glioma (Figure 8C), while it was positively associated with glioma grade, GGS riskscore, and glycolysis levels (Figure 8B,D,E). Comparing with NBTs, both mRNA and protein expression levels of ADM were significantly elevated in glioma tissues (Figure 8F,G). Among the four glioma cell lines studied, U251 and U373 cells exhibited the most pronounced upregulation of ADM mRNA levels (Figure 9A). Furthermore, the protein expression of ADM was also notably upregulated in U251 and U373 cells (Figure 9B).

We generated stable overexpression or knockdown cells to assess the influence of ADM on glycolysis and proliferation in glioma cells. We used qRT‐PCR and western blot assays to confirm the transfection efficiency (Figure 9C,D). Our findings revealed that ADM knockdown significantly reduced glucose consumption and lactate production in U251 and U373 cells (Figure 9E,F). To identify the ECAR, we employed Seahorse XF glycolytic stress assays. We discovered that ADM knockdown dramatically decreased the basal glycolysis and glycolytic capability in U251 and U373 cells (Figure 9G). Furthermore, cell viability assays demonstrated that ADM knockdown significantly inhibited U251 and U373 cell proliferation (Figure 9H). In contrast, ADM overexpression promoted glycolysis and proliferation in U251 and U373 cells (Figure 9E–H).

### 3.7 Identification of potential therapeutic agents for high‐GGS glioma patients

**FIGURE 10:**
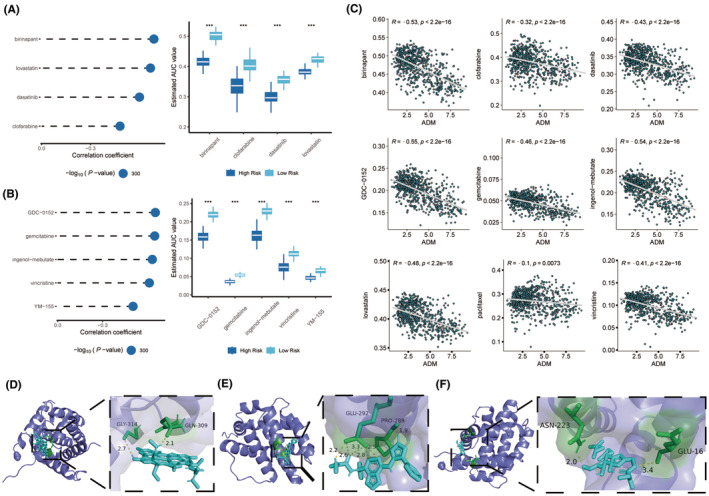
Drug sensitivity analysis and molecular docking. (A) The results of Spearman's correlation analysis and differential drug response analysis of four CTRP‐derived compounds. (B) The results of Spearman's correlation analysis and differential drug response analysis of five PRISM‐derived compounds. Note that lower values on the *y*‐axis of boxplots imply greater drug sensitivity. (C) The results of Spearman's correlation analysis of the expression of ADM and AUC value of 9 drugs. (D‐F) Schematic diagrams of the binding modes of representative component‐targets. (D) ADM with Birinapant; (E) ADM with GDC‐0152; (F) ADM with IM.

The CTRP v.2.0 and PRISM databases were utilized to identify potential therapeutic drugs for high‐risk GGS glioma patients. By analyzing the expression profiles of cell lines, the oncoPredict package was employed to predict drug sensitivity. As a result, nine compounds with lower AUC values were identified based on the thresholds of difference (log_2_FC >0.10) and correlation coefficients (Spearman's *R* < −0.30 for CTRP or Spearman's *R* < −0.35 for PRISM). These analyses yielded four CTRP‐derived compounds (including birinapant, lovastatin, dasatinib, and clofarabine) and five PRISM‐derived compounds (including GDC‐0152, gemcitabine, IM, vincristine, and YM‐155) (Figure 10A–C). The medications Birinapant (*R* = −0.53), GDC‐0152 (*R* = −0.55), and IM (*R* = −0.54) were deemed most likely to have a therapeutic effect in patients with high‐risk GGS glioma since they had the highest AUC values inversely correlated with ADM expression. To further determine which compound could bind with ADM, AutoDock and PyMOL software were used to predict and visualize the interaction of three compounds with ADM. The findings revealed that IM and ADM had excellent binding scores (affinity = −10.73 kcal/ mol) (Figure 10D–F; Table 1). Additionally, in vitro tests were conducted to observe the effect of IM in treating U251 and U373 cells with the upregulated expression of ADM.

**TABLE 1:** The selected compounds of docking results.

Target	Compound	Compound structure	Binding energy (kcal/mol)	Binding site
ADM	GDC‐0152	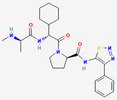	−7.71	ADM: A: PRO289:O ADM: A: GLU292:OE2
ADM	Birinapant	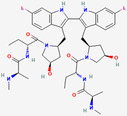	−4.27	ADM: A: GLN309:OE1
ADM	Ingenol_mebutate	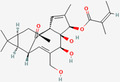	−10.73	ADM:A:ASN223:HD22

**FIGURE 11:**
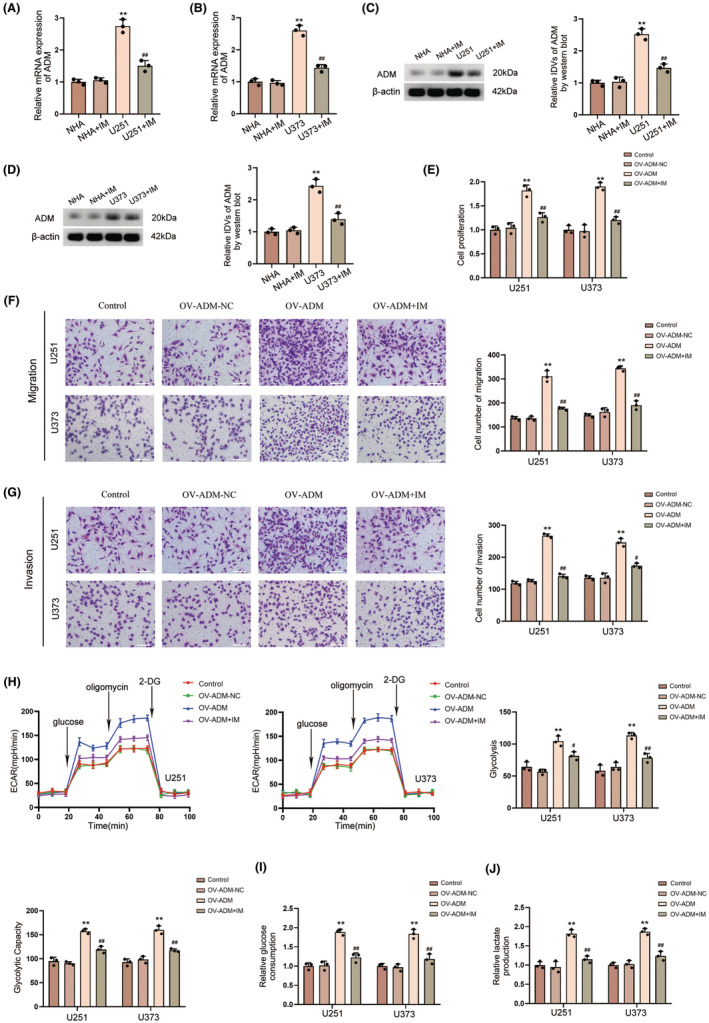
IM in vitro experiments. (A) PCR analysis showed the effect of IM among four groups (NHA, NHA+ IM, U251, U251+ IM). Data are shown as the mean ± SD (*n* = 3, each group). **p < 0.01 versus NHA group; ^##^ *p* < 0.01 versus U251 group. (B) PCR analysis showed the effect of IM among four groups (NHA, NHA+ IM, U373, U373+ IM). Data are shown as the mean ± SD (*n* = 3, each group). **p < 0.01 versus NHA group; ^##^ *p* < 0.01 versus U373 group. (C) Western blot analysis showed the effect of IM among four groups (NHA, NHA+ IM, U251, U251 + IM). Data are shown as the mean ± SD (*n* = 3, each group). **p < 0.01 versus NHA group; ^##^ *p* < 0.01 versus U251 group. (D) Western blot analysis showed the effect of IM among four groups (NHA, NHA+ IM, U373, U373+ IM). Data are shown as the mean ± SD (*n* = 3, each group). **p < 0.01 versus NHA group; ^##^ *p* < 0.01 versus U373 group. (E) The CCK‐8 assay showed the proliferation ability of U251 and U373 cells treated with IM. Data are shown as the mean ± SD (*n* = 3, each group). **p < 0.01 versus OV‐ADM‐NC group; ^##^ *p* < 0.01 versus OV‐ADM group. (F) The transwell assay was used to detect the migration ability of U251 and U373 cells treated with IM. Data are shown as the mean ± SD (*n* = 3, each group). **p < 0.01 versus OV‐ADM‐NC group; ^##^ *p* < 0.01 versus OV‐ADM group. (G) The transwell assay was used to detect the invasion ability of U251 and U373 cells treated with IM. Data are shown as the mean ± SD (*n* = 3, each group). **p < 0.01 versus OV‐ADM‐NC group; ^#^ *p* < 0.05, ^##^ *p* < 0.01 versus OV‐ADM group. (H) ECAR was measured to demonstrate the effects of IM. Data are shown as the mean ± SD (*n* = 3, each group). **p < 0.01 versus OV‐ADM‐NC group; ^#^ *p* < 0.05, ^##^ *p* < 0.01 versus OV‐ADM group. (I, J) The effects of IM on glucose consumption (I) and lactate production (J) in U251 and U373 cells. Data are shown as the mean ± SD (*n* = 3, each group). **p < 0.01 versus OV‐ADM‐NC group; ^##^ *p* < 0.01 versus OV‐ADM group.

We found that the expression of ADM in U251 and U373 cells treated with IM was significantly suppressed via qRT‐PCR and western blot experiments, while no significant change was observed in NHA cells (Figure 11A–D). The proliferation, invasion, and migration of U251 and U373 cells treated with IM were all significantly inhibited (Figure 11E–G). Similarly, the glucose consumption and lactate production, the basal glycolysis and glycolytic capability of U251 and U373 cells treated with IM were all significantly downregulated (Figure 11H–J).

## 4 DISCUSSION

To enhance the effectiveness of glioma therapy, we developed a robust glycolysis‐related subtyping system characterized by distinct clinical and molecular attributes. Notably, although the GS exhibited strong correlations with existing classifications, only a limited number of our classifier genes overlapped with the signatures of all prior classifications, indicating both substantial biological convergence and distinctive specializations. The GS taxonomy was meticulously validated across external datasets due to the importance of molecular subtype stability and reproducibility for therapeutic applications. Across all external datasets, the GS taxonomy exhibited analogous transcriptional and clinical features while maintaining comparable proportions. Furthermore, in addition to providing clear biological and molecular interpretations, the GS taxonomy also furnished a solid foundation for future clinical classification and therapeutic strategies.

GS1, a mixed subtype, exhibits moderate levels of stromal and immune activities. This subtype represents an intermediate state between GS2/5 and GS3/4. GS2, a mixed metabolic and immune subtype, is associated with glycolysis, amino sugar and nucleotide sugar metabolism, and humoral response. Metabolic inhibitors targeting glycolysis have been shown to be effective in treating GS2 tumors. Previous research has demonstrated that targeting key glycolytic proteins in glioma can trigger apoptosis.^23^ Due to enhanced immune activation, this subtype may achieve favorable clinical outcomes. Anti‐CTLA4 treatment has been observed to effectively treat GS2 tumors without requiring additional interventions. GS3, a KRAS‐activated subtype, is characterized by high tumor purity and immune‐desert. The lack of immunological storage may contribute to GS3's resistance to immunotherapy. The KRAS pathway can be directly targeted and is considered a proto‐oncogene in glioma.^24^, ^25^, ^26^ GS4 is characterized by high tumor purity, immune, and stromal‐desert. Furthermore, immune‐desert TME in GS4 tumors explains why these malignancies logically develop resistance to immunotherapy. The abnormal activation of the hedgehog signaling pathway is associated with the development of GS4 tumors. Previous studies^27^, ^28^, ^29^, ^30^ have demonstrated that the activation of this pathway in glioma patients results in the expression of malignant characteristics.

GS5, an immunologically activated subtype, is linked to elevated infiltration of macrophages M2, cancer‐associated fibroblasts (CAFs), endothelial cells, Th2 cells, and Th1 cells. GS5 exhibits significant enrichment of cell cycle and DNA repair pathways. GS5 exhibit a favorable response to anti‐PD1 therapy. Despite its high immune cell infiltration, the GS2/5 subtype has a poor prognosis due to the predominance of immunosuppressive cells, including fibroblasts, Treg cells, and M2 macrophages, which results in impaired immune function.

Metabolic reprogramming and migration, invasion, are tightly interconnected rather than independent.^31^ Glycolysis promotes the generation of acids in tumor cells,^32^ leading to extracellular matrix degradation through protein hydrolytic enzymes, thereby enhancing the migratory and invasive abilities of tumor cells.^33^, ^34^ Glycolysis is crucial for glioma to exhibit malignant phenotype. Targeting glycolysis‐associated proteins, such as lactate dehydrogenase A (LDHA), glucose transporter type 1 (GLUT1), and pyruvate kinase M2 (PKM2), may impede the migration and invasion of glioma cells.^35^, ^36^, ^37^

ADM, a potent vasodilator peptide consisting of 52 amino acids, has been observed to be expressed in various human tumor cell lines including those of the lung, breast, brain, prostate, and colon. Its presence has been linked to tumor cell proliferation and angiogenesis.^38^, ^39^, ^40^, ^41^ Furthermore, ADM mRNA expression levels in brain tumors are positively correlated with tumor grade.^42^ Our research findings indicate that the suppression of ADM expression leads to a decrease in glycolysis, migration, invasion, and proliferation of glioma cells. Moreover, ingenol mebutate (IM), a hydrophobic diterpene ester derived from the plant Euphorbia peplus, has been approved for the treatment of skin malignancies.^43^ IM has demonstrated antitumor activity in leukemia, colon cancer, and melanoma cells by acting as a protein kinase C (PKC) agonist.^44^, ^45^ IM, identified as the most effective medication for the treatment of ADM, was discovered through a combination of drug sensitivity analysis, ADM expression analysis, and molecular docking. In vitro experiments confirmed that IM inhibited glioma cell proliferation, migration, and invasion while also suppressing ADM expression in U373 and U251 cells. This provides a novel and effective compound for the treatment of glioma in the future.

Our study, from a glycolysis perspective, identified and validated a high‐resolution taxonomy that could aid glioma patients in making informed decisions. The unique biology and clinical characteristics of the GS taxonomy enhance our understanding of glioma heterogeneity, facilitating patient stratification and individualized care management. Furthermore, IM and inhibiting ADM may offer more targeted interventions for glioma patients, which require further validation in clinical practice.

To further support the biological and clinical interpretability of novel GS taxonomy, a prospective multicenter investigation is still essential. In summary, we posit that this innovative high‐resolution GS taxonomy, IM, and ADM inhibition could enhance the efficacy of glioma treatment.

## 5 CONCLUSION

This study demonstrated that assessing GS would help us understand the heterogeneity of glioma, which was critical for predicting immune cell infiltration (ICI) characterization, prognosis, and customized immunotherapy treatments. We further explored the glycolysis‐related gene ADM and IM to provide a theoretical framework for anti‐tumor strategy targeting glycolysis.

## AUTHOR CONTRIBUTIONS

Shaolong E had full access to all the data in the study and takes responsibility for the integrity of the data and the accuracy of the data analysis. Shaolong E: Protocol/project development; Tianshu Ying, Yaming Lai, Shiyang Lu, and Shaolong E: Bioinformatics analysis and graphing; Tianshu Ying, Yaming Lai, Shiyang Lu, and Shaolong E: In vitro experiments and data analysis; Tianshu Ying and Shaolong E: Manuscript writing/editing.

## CONFLICT OF INTEREST STATEMENT

Tianshu Ying, Yaming Lai, Shiyang Lu, and Shaolong E declare that they have no competing interests.

## Supporting information


Data S1.

Table S1.
Table S2.
Table S3.

Figure S1.

## Data Availability

The data that support the findings of this study are available from the corresponding author upon reasonable request.
