# Supplementary material for: Identification and validation of a glycolysis‐related taxonomy for improving outcomes in glioma
Source: CNS Neurosci Ther. 2024 Feb 8;30(2):e14601. doi: 10.1111/cns.14601 (PMC10853657; doi:10.1111/cns.14601)

Full unedited blot for Figure 8F

ADM

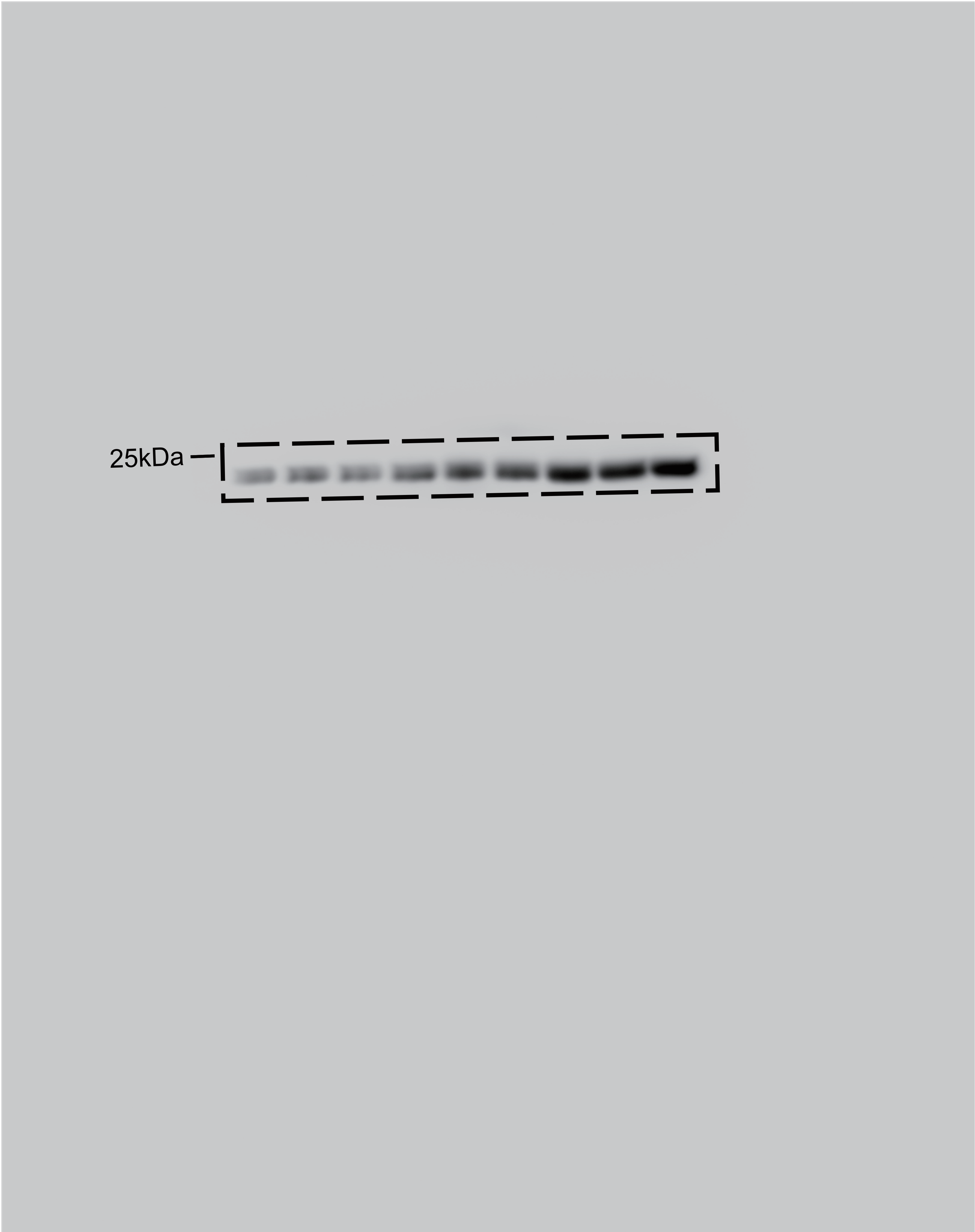

β-actin

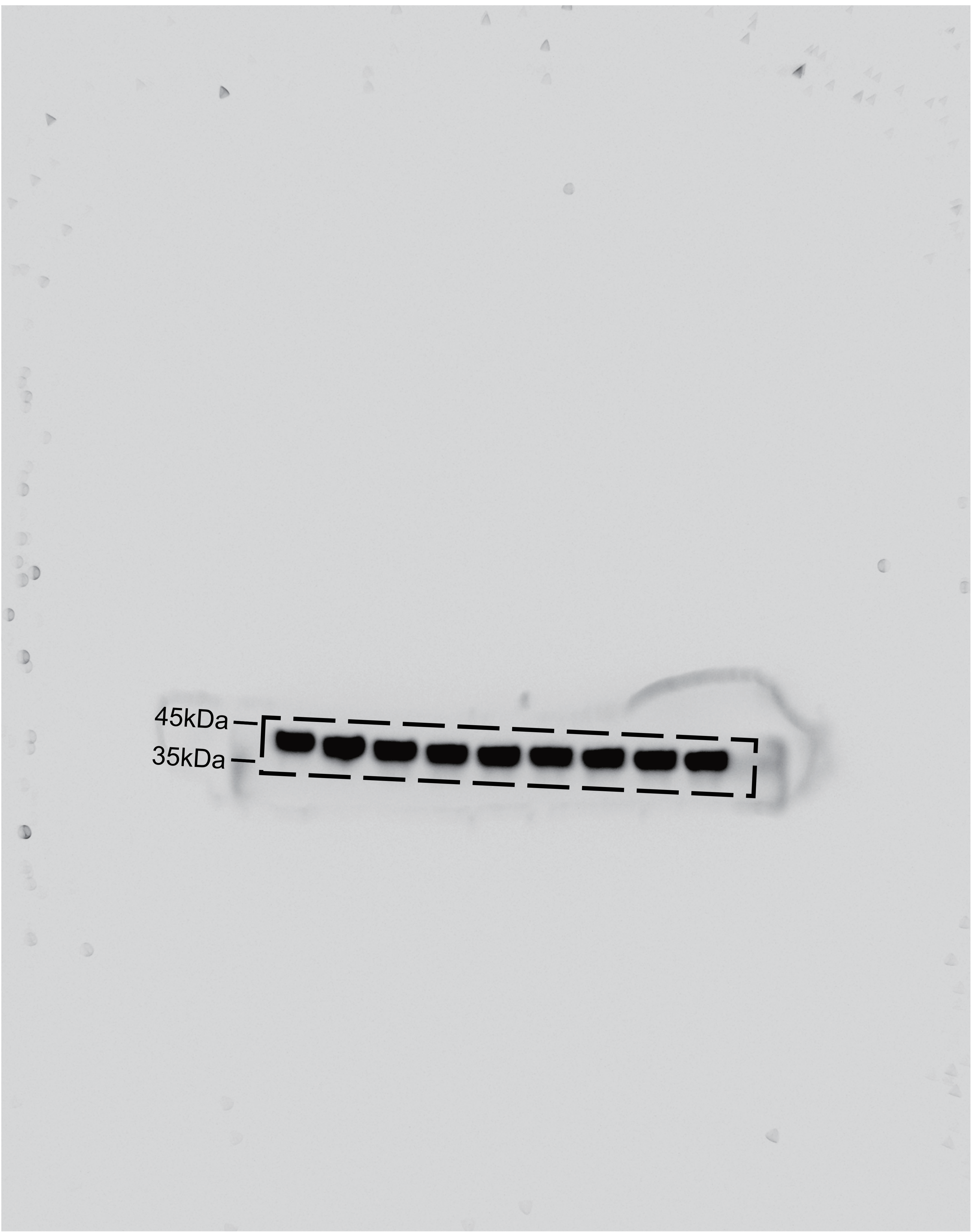

Full unedited blot for Figure 9B

ADM

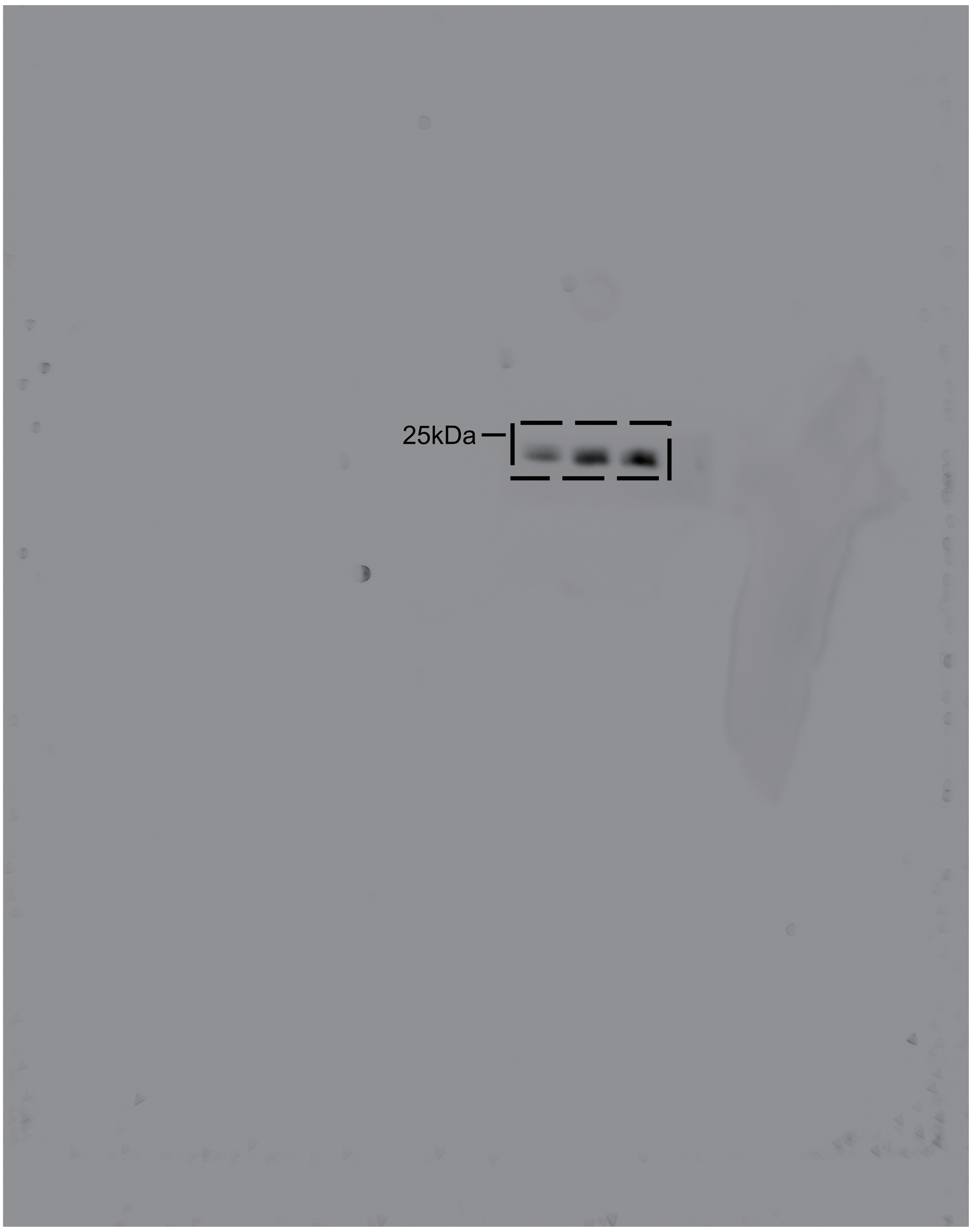

β-actin

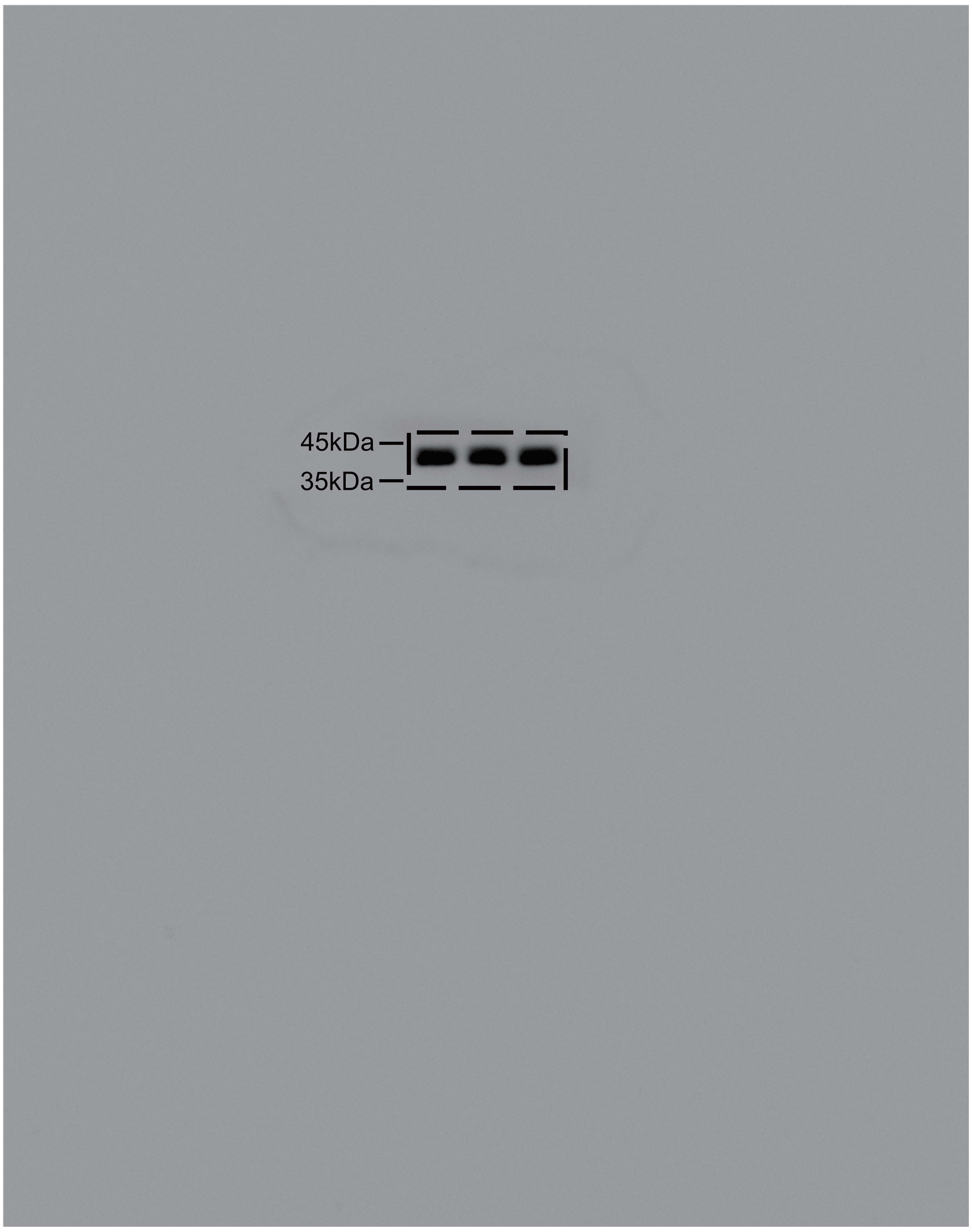

Full unedited blot for Figure 9D-U251

ADM

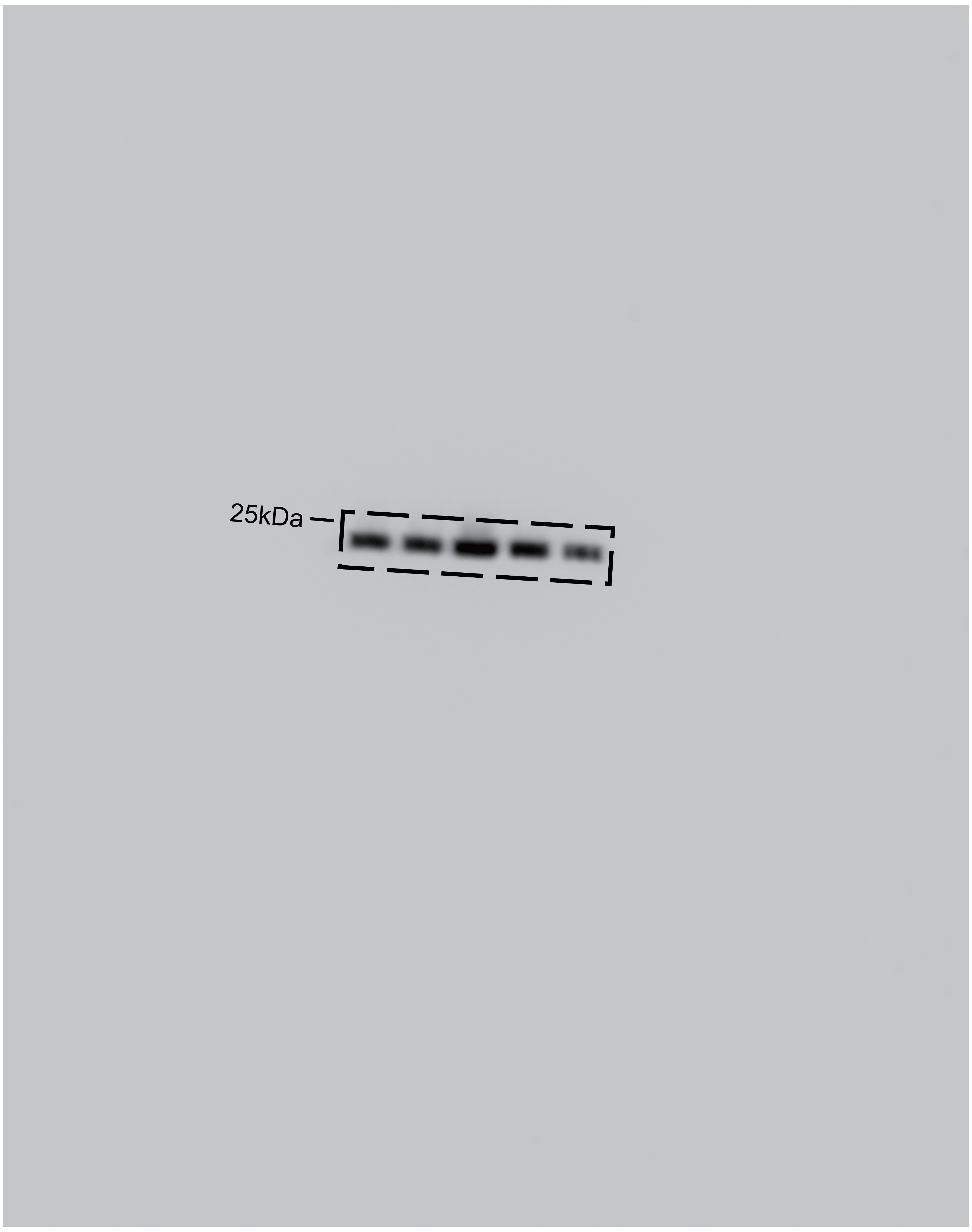

β-actin

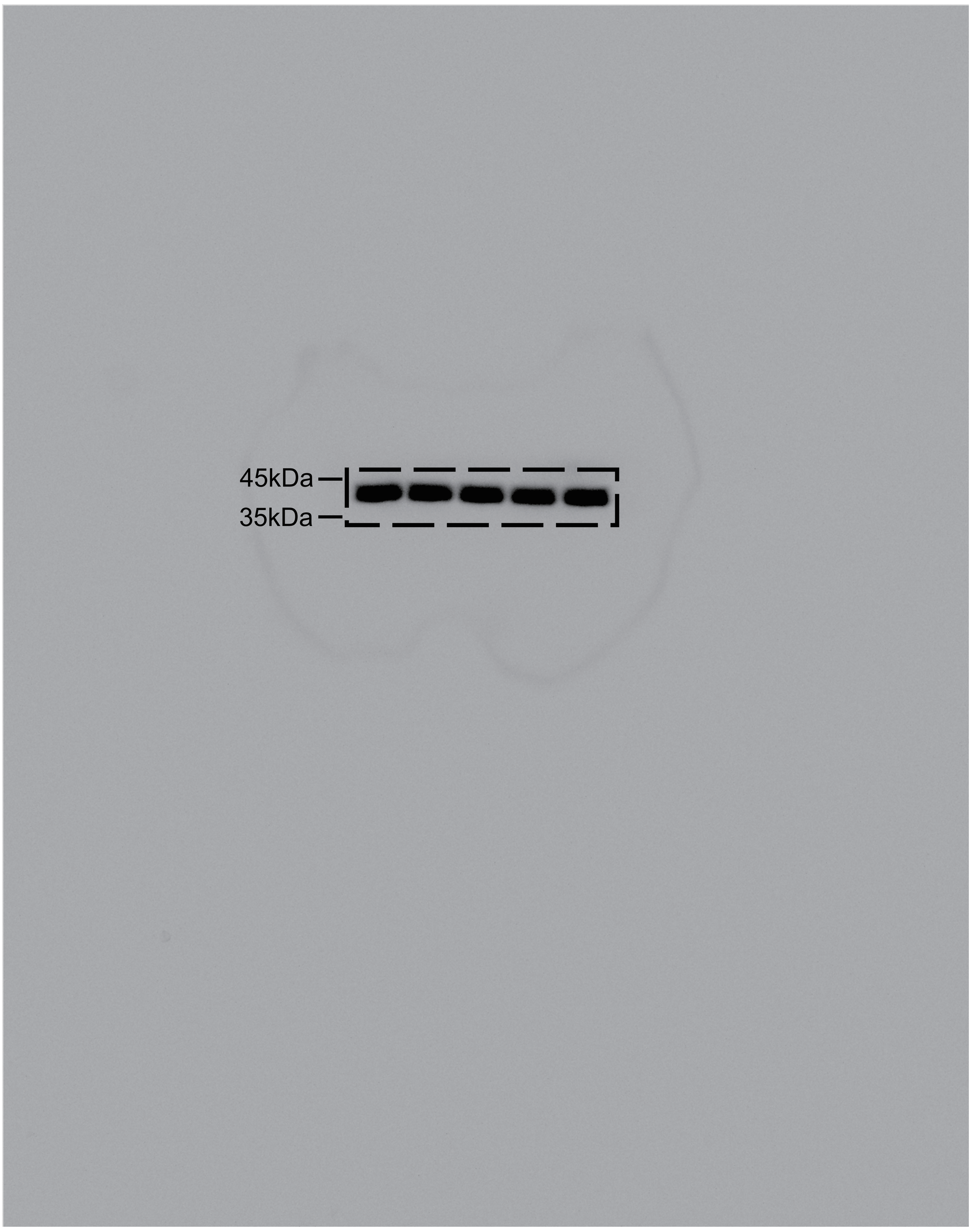

Full unedited blot for Figure 9D-U373

ADM

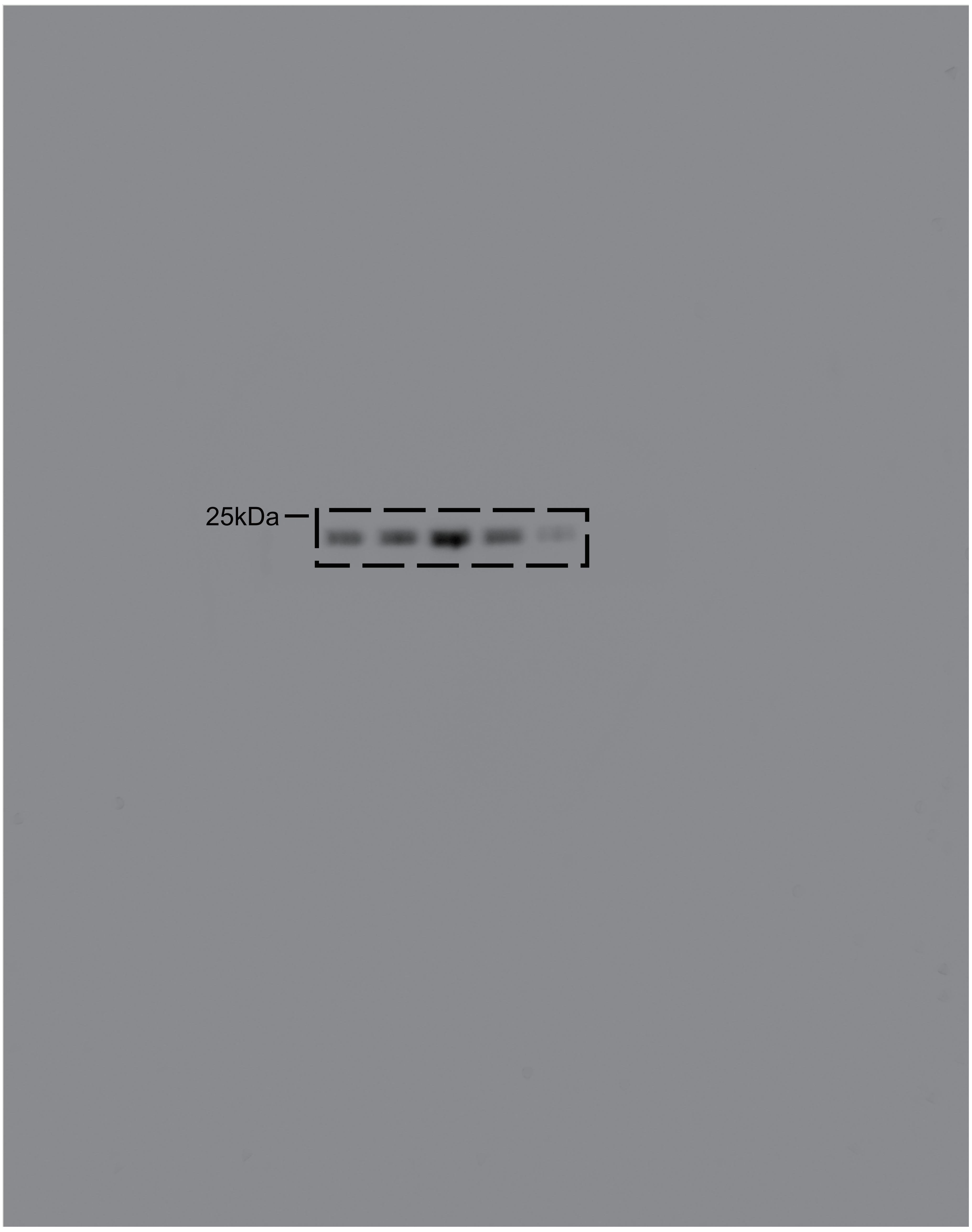

β-actin

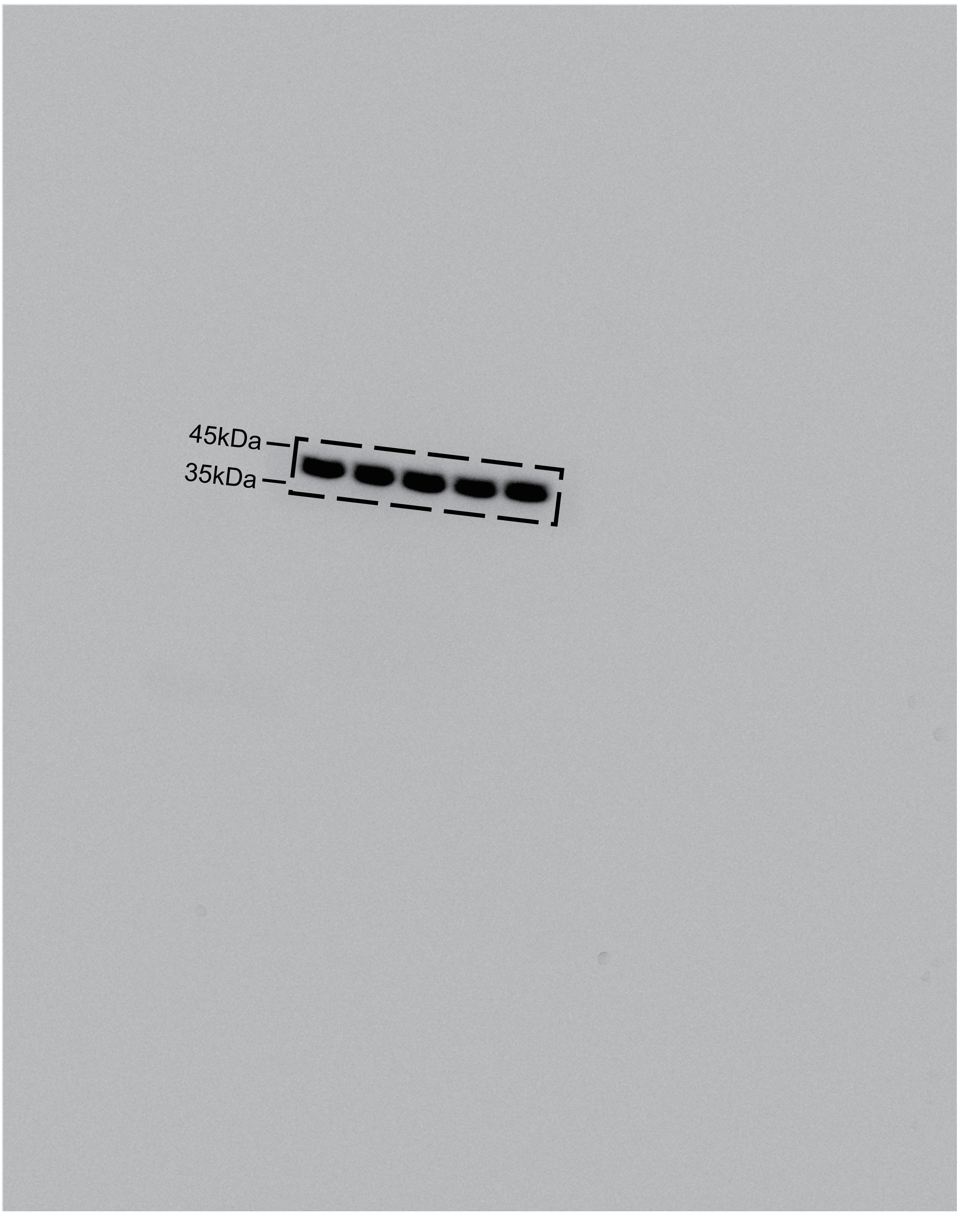

Full unedited blot for Figure 11C

ADM

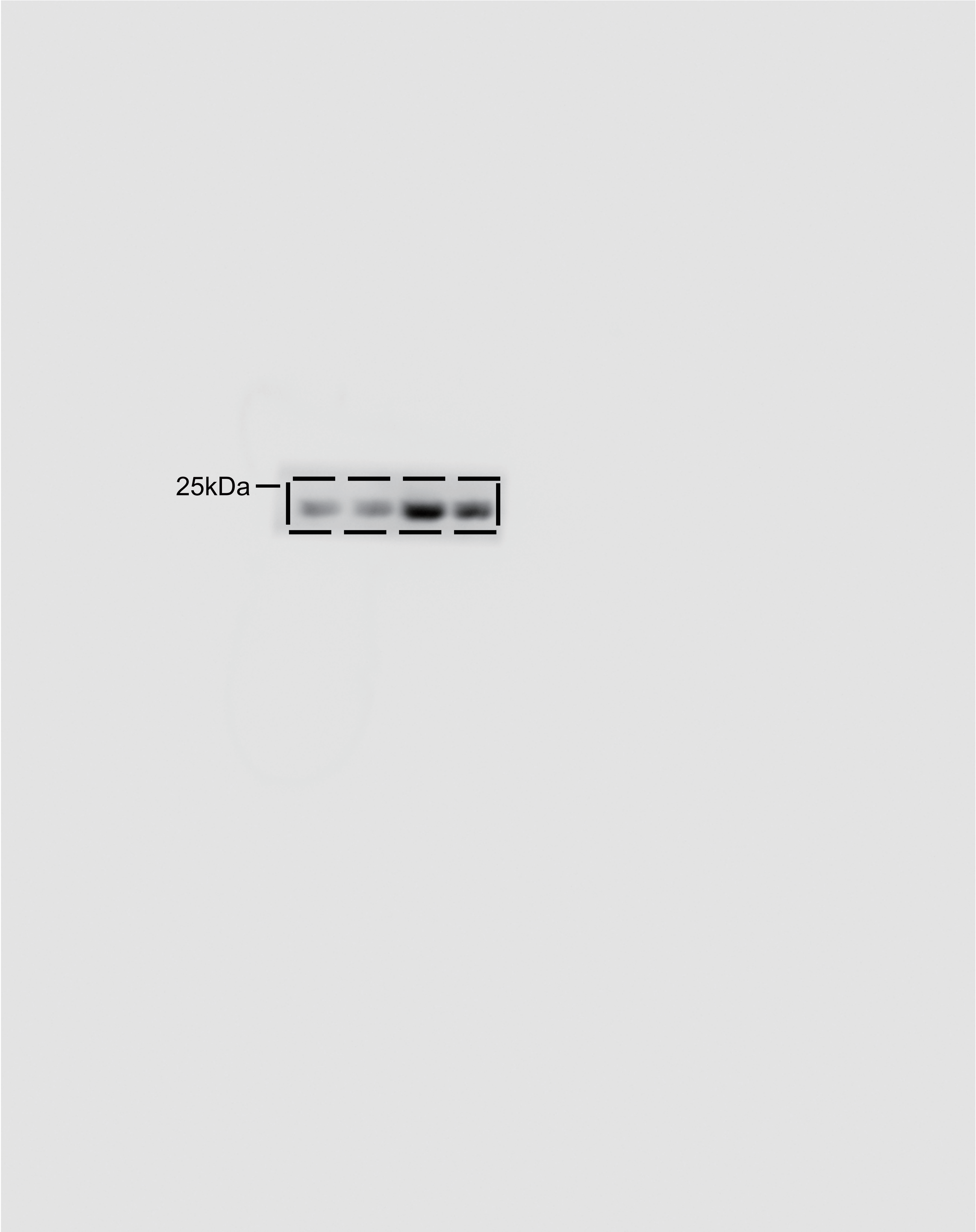

β-actin

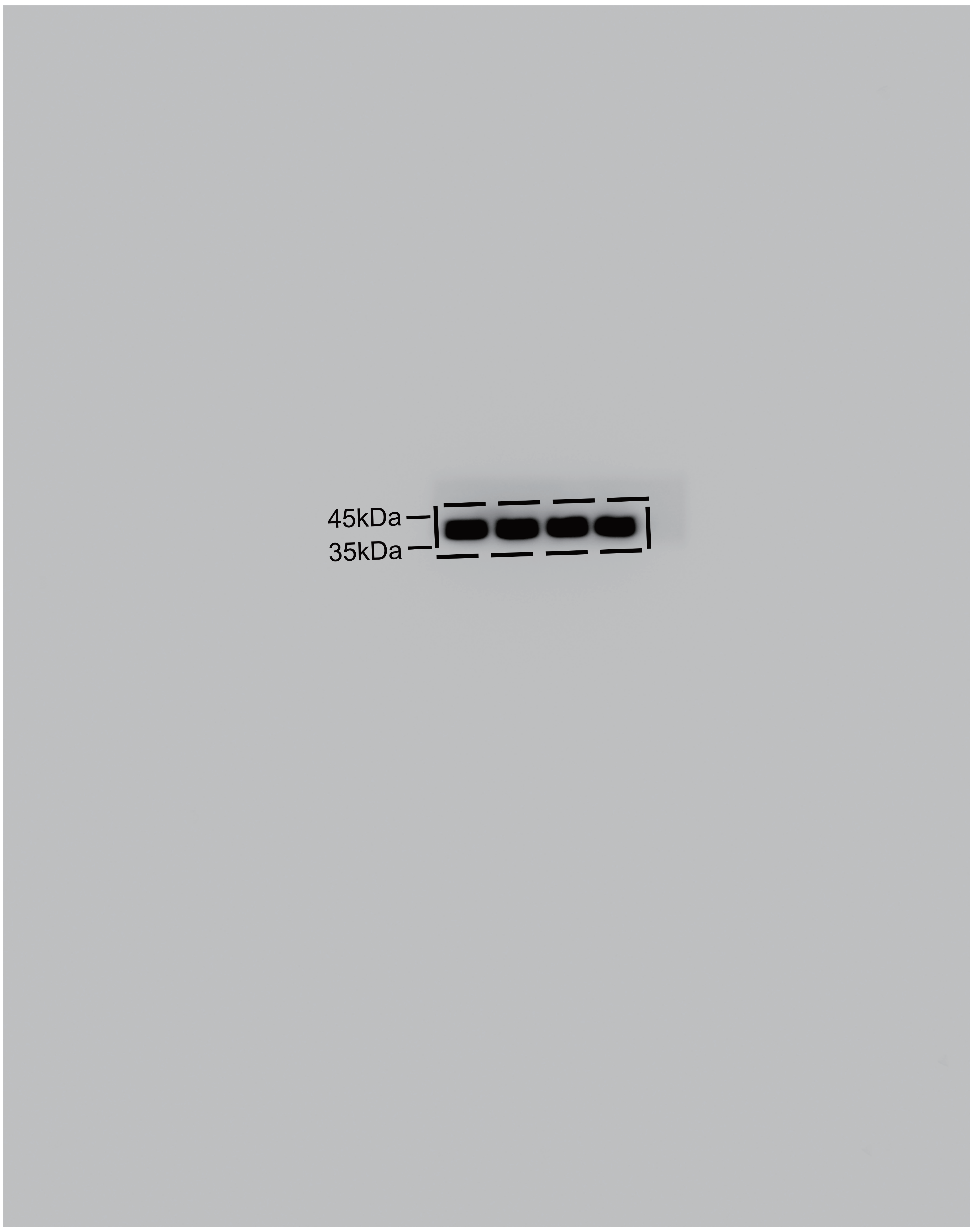

Full unedited blot for Figure 11D

ADM

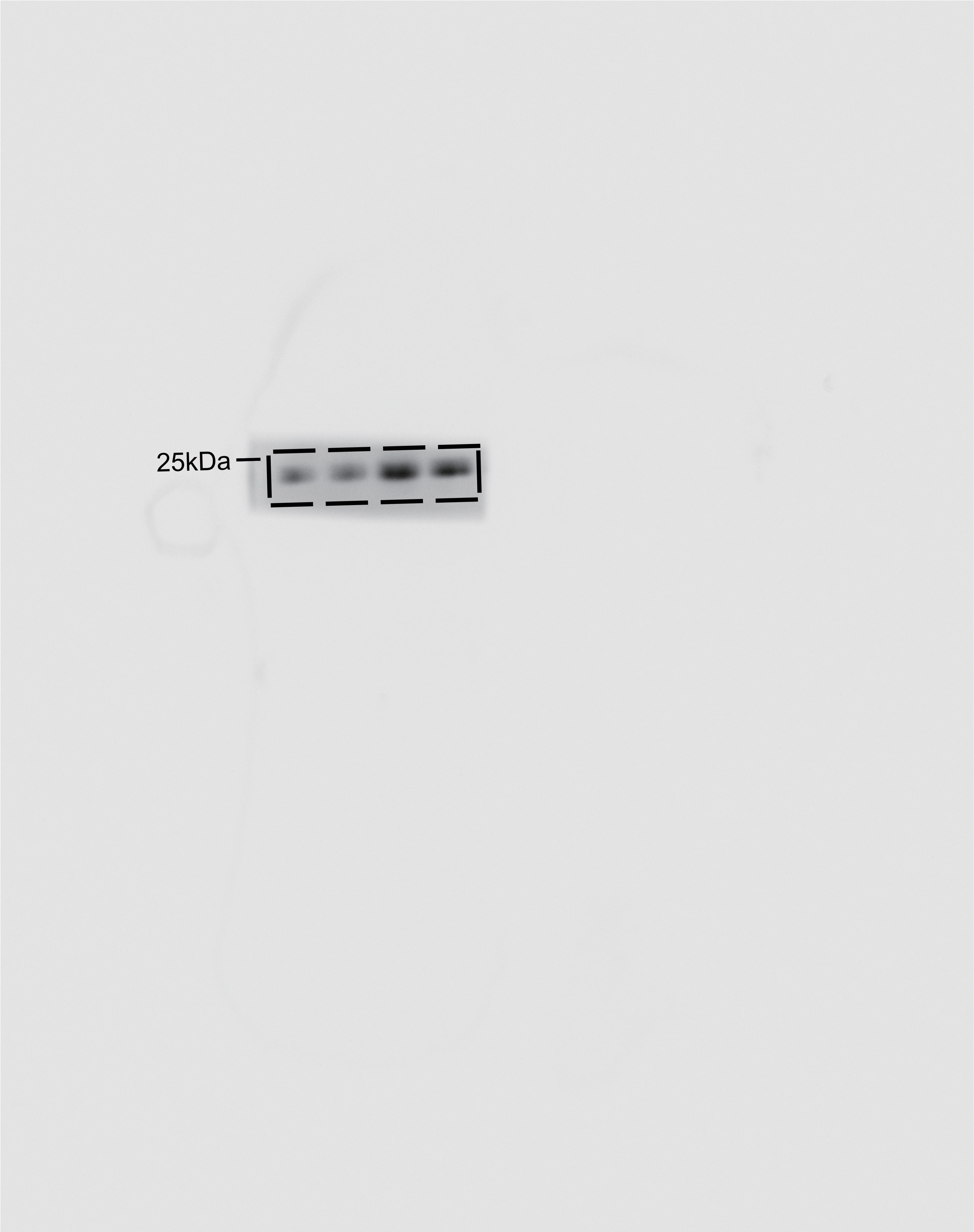

β-actin

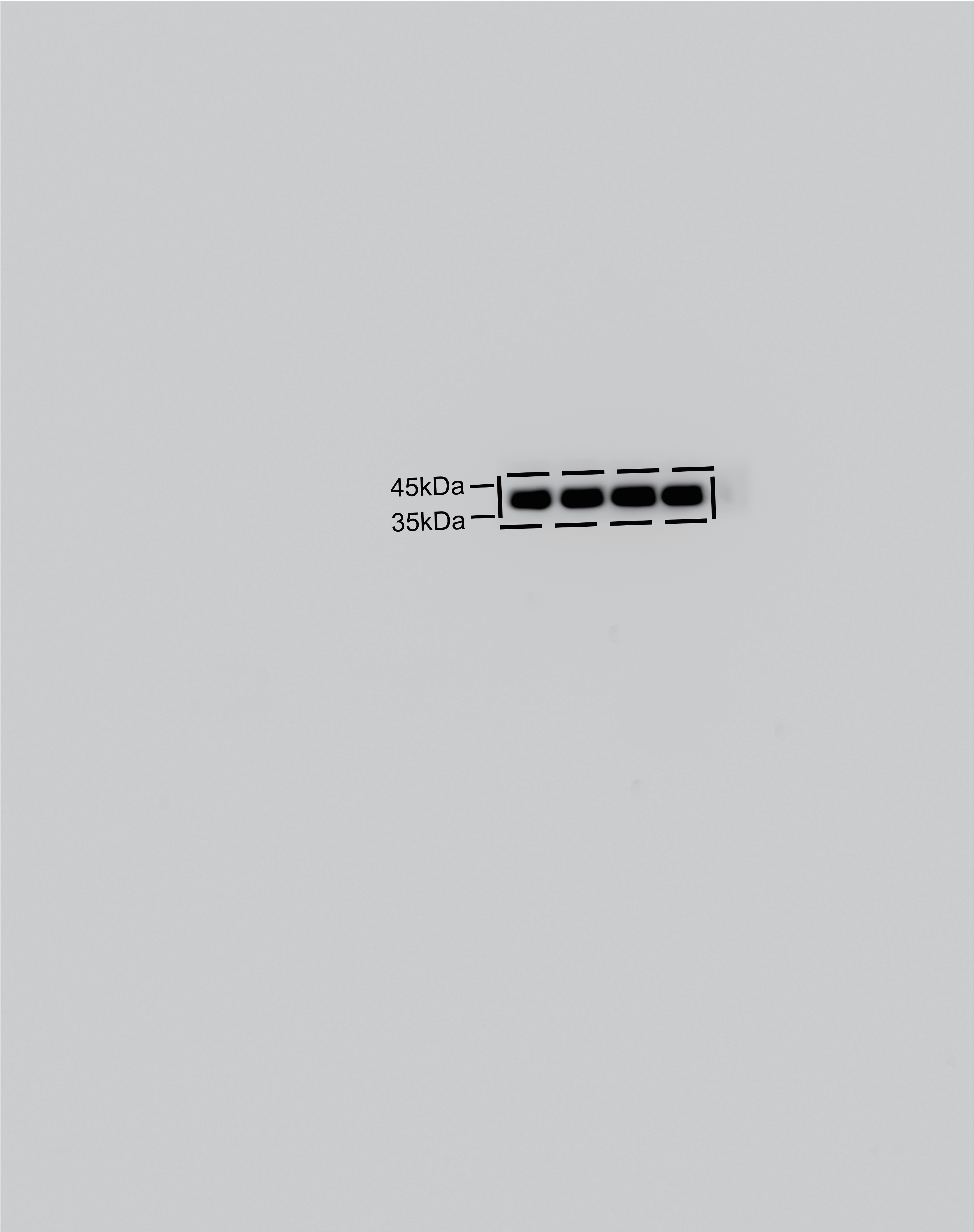

Supplement: Supplementary file 1 — Data S1. [file CNS-30-e14601-s001.pdf]
