# Supplementary material for: Identification and validation of a glycolysis‐related taxonomy for improving outcomes in glioma
Source: CNS Neurosci Ther. 2024 Feb 8;30(2):e14601. doi: 10.1111/cns.14601 (PMC10853657; doi:10.1111/cns.14601)
Supplement: Supplementary file 2 — Table S1. Table S2. Table S3. [file CNS-30-e14601-s003.docx]

Supplementary Table 1. Website

| Database | Website |
| --- | --- |
| UCSC | https://xenabrowser.net/datapages/ |
| CGGA | http://www.cgga.org.cn/ |
| GSEA | https://www.gsea-msigdb.org/gsea/msigdb/index.jsp |
| GlioVis | http://gliovis.bioinfo.cnio.es/ |
| PRISM | https://depmap.org/portal/prism/ |
| CTRP v.2.0 | https://portals.broadinstitute.org/ctrp |

Supplementary Table 2. The short hairpin RNAs and their sequences

| gene | Site | Sequence (5’-3’) |
| --- | --- | --- |
| ADM | #1 | GATCTACCAGTTCACAGATAA |
|  | #2 | GTTTCGAAAGAAGTGGAATAA |
|  | #3 | CCCACTTTCTTTAGGATTTAG |

Supplementary Table 3. The primers for qRT–PCR assays.

| Gene | Primer | Sequence |
| --- | --- | --- |
| ADM | Forward | GATGTACCTGGGTTCGCTCG |
|  | Reverse | CTCTTCCCACGACTCAGAGC |
| β-actin | Forward | ACTTCGAGCAAGAGATGGCC |
|  | Reverse | CCAGGAAGGAAGGCTGGAAG |
