# Supplementary figures and images for: Identification and validation of a glycolysis‐related taxonomy for improving outcomes in glioma
Source: CNS Neurosci Ther. 2024 Feb 8;30(2):e14601. doi: 10.1111/cns.14601 (PMC10853657; doi:10.1111/cns.14601)

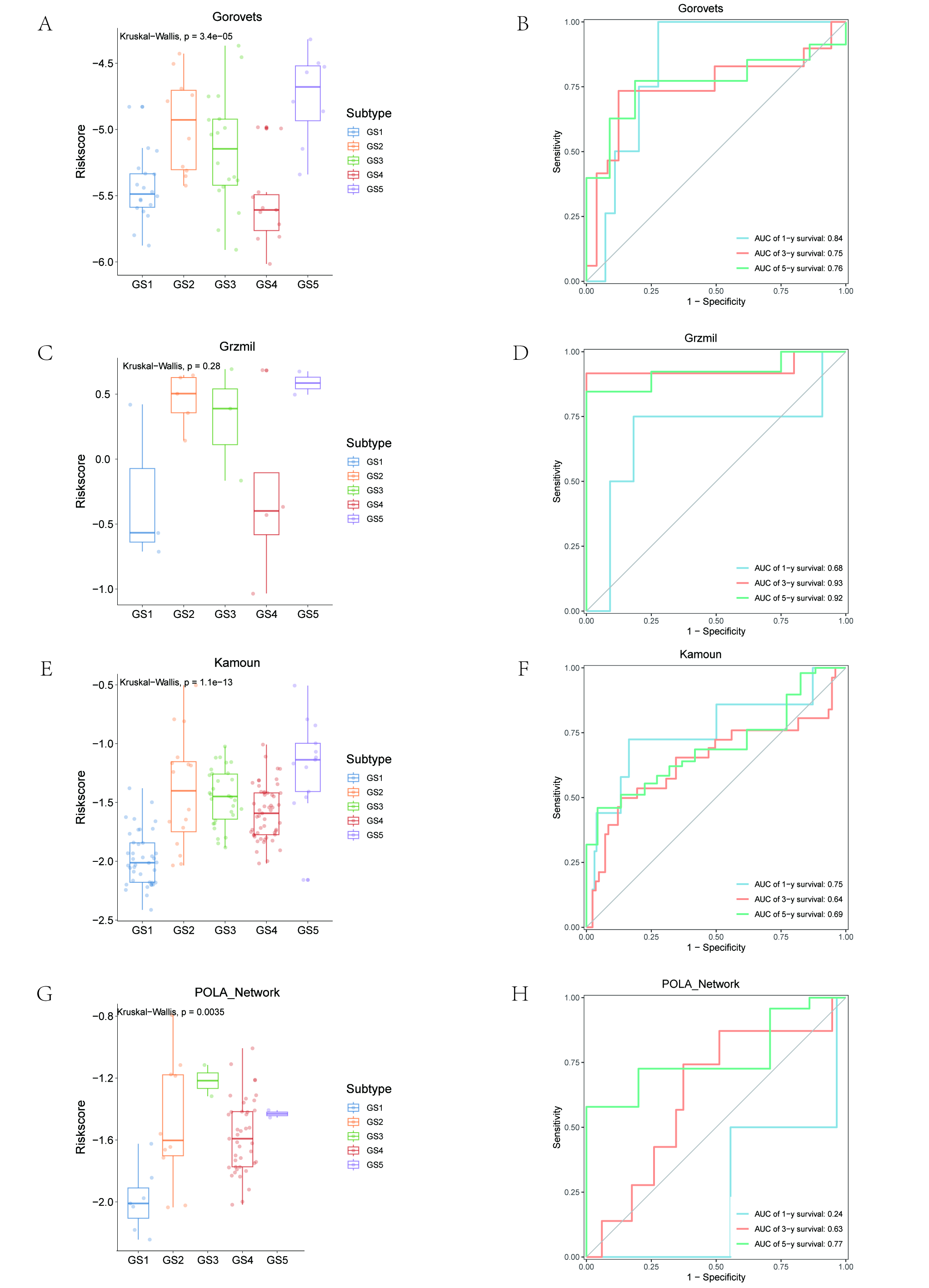

Supplement: Supplementary file 3 — Figure S1. [file CNS-30-e14601-s002.tif]
